# Prediction of Graft-Versus-Host Disease in Humans by Donor Gene-Expression Profiling

**DOI:** 10.1371/journal.pmed.0040023

**Published:** 2007-01-30

**Authors:** Chantal Baron, Roland Somogyi, Larry D Greller, Vincent Rineau, Peter Wilkinson, Carolyn R Cho, Mark J Cameron, David J Kelvin, Pierre Chagnon, Denis-Claude Roy, Lambert Busque, Rafick-Pierre Sékaly, Claude Perreault

**Affiliations:** 1 Institute of Research in Immunology and Cancer, University of Montreal, Montreal, Quebec, Canada; 2 Department of Medicine, University of Montreal, Montreal, Quebec, Canada; 3 Division of Hematology, Maisonneuve-Rosemont Hospital, Montreal, Quebec, Canada; 4 Biosystemix Limited, Sydenham, Ontario, Canada; 5 Lady Davis Institute for Medical Research, Montreal, Quebec, Canada; 6 Toronto General Research Institute, Toronto, Ontario, Canada; 7 Centre de Recherche du Centre Hospitalier de l'Université de Montréal, Montréal, Quebec, Canada; Fred Hutchison Cancer Research Center, United States of America

## Abstract

**Background:**

Graft-versus-host disease (GVHD) results from recognition of host antigens by donor T cells following allogeneic hematopoietic cell transplantation (AHCT). Notably, histoincompatibility between donor and recipient is necessary but not sufficient to elicit GVHD. Therefore, we tested the hypothesis that some donors may be “stronger alloresponders” than others, and consequently more likely to elicit GVHD.

**Methods and Findings:**

To this end, we measured the gene-expression profiles of CD4^+^ and CD8^+^ T cells from 50 AHCT donors with microarrays. We report that pre-AHCT gene-expression profiling segregates donors whose recipient suffered from GVHD or not. Using quantitative PCR, established statistical tests, and analysis of multiple independent training-test datasets, we found that for chronic GVHD the “dangerous donor” trait (occurrence of GVHD in the recipient) is under polygenic control and is shaped by the activity of genes that regulate transforming growth factor-β signaling and cell proliferation.

**Conclusions:**

These findings strongly suggest that the donor gene-expression profile has a dominant influence on the occurrence of GVHD in the recipient. The ability to discriminate strong and weak alloresponders using gene-expression profiling could pave the way to personalized transplantation medicine.

## Abstract 2

The donor gene expression profile appears to have a dominant influence on the occurrence of graft-versus-host disease in the recipient.

## Editors' Summary

**Background.:**

Human blood contains red blood cells, white blood cells, and platelets, which carry oxygen throughout the body, fight infections, and help blood clot, respectively. Normally, blood-forming (hematopoietic) stem cells in the bone marrow (and their offspring, peripheral blood stem cells) continually provide new blood cells. Tumors that arise from the bone marrow (such as leukemia and lymphoma, two types of hematopoietic tumor) are often treated by a bone marrow or peripheral blood stem cell transplant from a healthy donor to provide new blood-forming stem cells, as a follow-up to chemotherapy or radiotherapy designed to eradicate as much of the tumor as possible. This procedure is called allogeneic hematopoietic cell transplantation (AHCT)—the word allogeneic indicates that the donor and recipient are not genetically identical. When solid organs (for example, kidneys) are transplanted, the recipient's immune system can recognize alloantigens (proteins that vary between individuals) on the donor organ as foreign and reject it. To reduce the risk of rejection, the donor and recipient must have identical major histocompatibility complex (MHC) proteins. MHC matching is also important in AHCT but for further reasons. Here, donor T lymphocytes (a type of white blood cell) can attack the skin and other tissues of the host. This graft versus host disease (GVHD) affects many people undergoing AHCT despite MHC matching either soon after transplantation (acute GVHD) or months later (chronic GVHD). As an aside, the transplant may also act against the tumor itself—this is known as a graft versus leukemia effect.

**Why Was This Study Done?:**

GVHD can usually be treated with drugs that damp down the immune system (immunosuppressive drugs), but it would be preferable to avoid GVHD altogether. Indeed, GVHD continues to be the leading cause of nonrelapse mortality following AHCT. Unfortunately, what determines who will develop GVHD after MHC-matched AHCT is unclear. Although GVHD only develops if there are some mismatches in histocompatibility antigens between the donor and host, it does not inevitably develop. Until now, scientists have mainly investigated whether differences between ACHT recipients might explain this observation. But, in this study, the researchers have examined the donors instead to see whether differences in their immune responses might make some donors stronger “alloresponders” than others and consequently more likely to cause GVHD.

**What Did the Researchers Do and Find?:**

The researchers used a molecular biology technique called microarray expression profiling to examine gene expression patterns in the T lymphocytes of peripheral blood stem cell donors. From these patterns, they identified numerous genes whose expression levels discriminated between donors whose MHC-identical transplant recipient developed GVHD after AHCT (GVHD^+^ donors) and those whose recipient did not develop GVHD (GVHD^−^ donors). The researchers confirmed that the expression levels of 17 of these genes discriminated between GVHD^+^ and GVHD^−^ donors using a second technique called quantitative reverse transcriptase polymerase chain reaction. Many of these genes are involved in TGF-β signaling (TGF-β is a protein that helps to control the immune system), cell growth, or proliferation. The researchers also identified four gene pairs that interacted with each other to determine the likelihood that a given donor would induce GVHD. Finally, the researchers computationally retested their data and showed that the measurement of expression levels of each of these genes and of the four interacting gene pairs could correctly identify a donor sample likely to cause GVHD in up to 80% of samples.

**What Do These Findings Mean?:**

These findings provide the first evidence that the donor's gene expression profile influences the development of GVHD in the recipient after AHCT. The researchers suggest that a “dangerous donor” (strong alloresponder) is a key factor in determining whether GVHD occurs after AHCT and propose that gene expression profiling of donor T lymphocytes might identify those donors likely to cause GVHD. Before this approach can be used to reduce the incidence of GVHD after AHCT, these findings need to be confirmed in many more donors. Also, the development of a test that is accurate enough for clinical use—one that does not miss dangerous donors but does not discard too many safe donors—may require the identification of larger groups of interacting genes. But, if it survives further investigation, the concept of a dangerous donor could represent an important advance in transplantation medicine, one that could help clinicians select low-risk donors for AHCT and tailor patients' immunosuppressive drug regimens according to their donor-determined risk of GVHD.

**Additional Information.:**

Please access these Web sites via the online version of this summary at http://dx.doi.org/doi:10.1371/journal.pmed.0040023.

• The National Marrow Donor Program provides information for patients and physicians on all aspects of hematopoietic stem cell transplantation, including GVHD

• The MedlinePlus encyclopedia has pages on bone marrow transplants, GVHD and transplant rejection

• The US National Cancer Institute has a factsheet on bone marrow and peripheral blood stem cell transplantation

## Introduction

Graft-versus-host disease (GVHD) is initiated by donor T cell responses to host alloantigens [1–3]. However, the occurrence and severity of GVHD are not determined solely by the level of histoincompatibility between donor and recipient. Thus, two major histocompatibility complex (MHC)-identical individuals (excluding identical twins) or two inbred strains of mice will display over 50 minor histocompatibility antigen differences [4,5]. If histoincompatibility was sufficient for triggering GVHD, the rate of GVHD in MHC-matched recipients of allogeneic hematopoietic cell transplantation (AHCT) that receive no immunosuppressive agents should therefore be 100%. Under these conditions, however, GVHD was found in only 50% and 73% of mouse and human recipients, respectively [6,7]. Even in mouse MHC-mismatched AHCT models, some, but not all, donor strains induce severe acute GVHD (aGVHD) [8,9]. Thus, histoincompatibility is necessary, but not sufficient, to elicit fatal GVHD. Recent evidence suggests that aside from the mere presence of genetic polymorphisms, two host factors may influence the severity of aGVHD and chronic GVHD (cGVHD): elusive properties (for example, tissue distribution) of the immunodominant host alloantigens [10] and polymorphisms of host cytokine genes [11,12]. Another nonexclusive and largely unexplored rationale would be that some donors are “stronger alloresponders” than others because of quantitative or qualitative differences in immune responses. Indirect evidence for the latter hypothesis are reports suggesting that several donor genetic polymorphisms may correlate with GVHD severity [12].

The seminal studies of Biozzi and colleagues have shown that the strength of B cell responses to natural immunogens is under multigenic control [13,14]. Approximately ten independently segregating loci endowed with additive effects are responsible for the major (240-fold) multispecific differences separating high- and low-antibody responders [15,16]. No similar data are available for T cell responses in general, and those against histocompatibility antigens in particular. Since GVHD is by far the main barrier in AHCT [17–20], identification of high-risk donors would allow better donor selection and tailoring of immunosuppressive regimens to GVHD risk. In addition to complex genetic trait linkages, it may also be assumed that environmental factors and donor immune system histories may contribute toward determining GVHD. While the latter two factors would be hidden from the analysis of inherited genetic traits or gene-sequence variation, they might be reflected in gene-expression signatures. We therefore chose to measure the activity of a broad range of genes with expression microarrays as a means of surveying the overall molecular-state signature of the donor immune system, independent of whether that state is largely determined by inherited genetic factors, environment, donor history, or mixtures thereof. The objective of our study was, therefore, to determine whether gene-expression profiling could discriminate AHCT donors that induced either aGVHD or cGVHD in their recipient host from donors who did not. In other words, is it possible to distinguish high from low alloresponders? Notwithstanding the fundamental importance of that question, a positive answer could pave the way to personalized transplantation medicine.

## Methods

### Study Patients

Only patients with hematological malignancies and their healthy sibling donors who were identical with regard to HLA participated in this study (Table 1). The AHCT myeloablative regimen consisted of cyclophosphamide (120 mg/kg) and total body irradiation (12 Gy), or busulfan (16 mg/kg) and cyclophosphamide (200 mg/kg). All patients received unmanipulated peripheral blood–stem-cell grafts (mobilized with G-CSF) and were given GVHD prophylaxis consisting of cyclosporine A and short-course methotrexate [21]. Donor blood samples were obtained one day prior to mobilization of peripheral blood–progenitor cells with G-CSF. Diagnosis of aGVHD and cGVHD was made after clinical evaluation and histologic confirmation according to previously reported criteria [22–24]. Patients with grade 0 and grades I–IV aGVHD were considered aGVHD− and +, respectively. Biopsies of skin and gut were carried out in 90% and 15% of patients with aGVHD, respectively; overall, 95% of participants with aGVHD had biopsies, including all patients with grade I GVHD. All participants with cGVHD showed extensive clinical GVHD [19]. Clinical protocols were approved by the Human Subjects Protection Committee of the Maisonneuve-Rosemont Hospital. Samples were obtained with the informed consent of the patients.

**Table 1:**
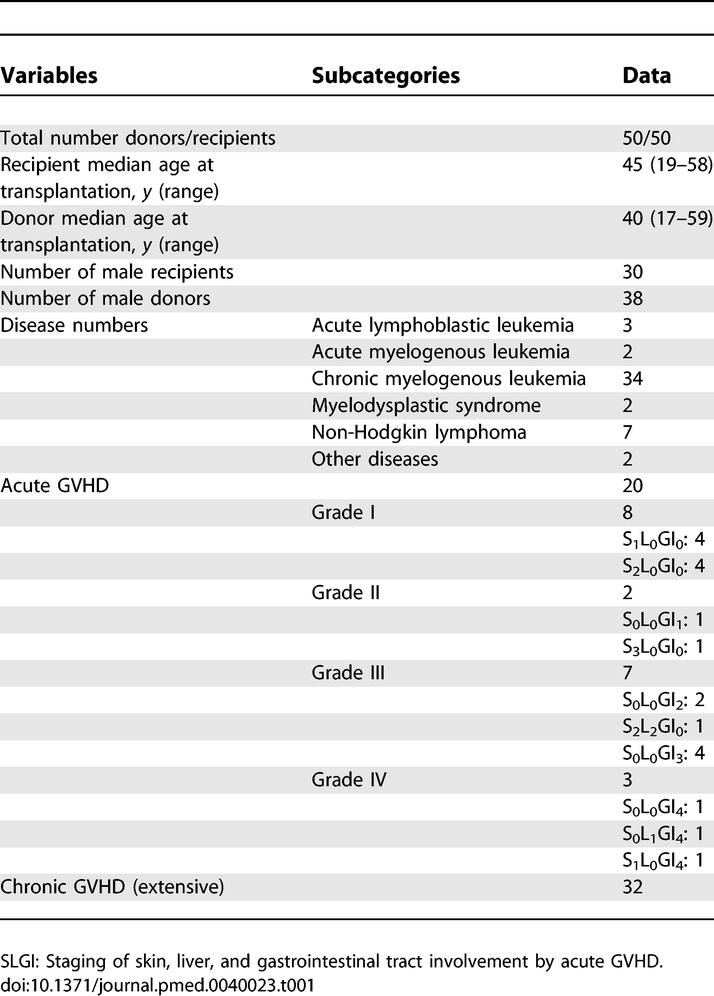
Donor–Recipient Characteristics

### RNA Isolation, Amplification, and Microarray Hybridization

CD4^+^ and CD8^+^ T cells were enriched from peripheral blood mononuclear cells by positive isolation using magnetic microbeads (Dynal, http://www.invitrogen.com). Sample RNA was extracted using an RNA extraction kit (Qiagen, http://www.qiagen.com), then amplified using the MessageAmp RNA kit (Ambion, http://www.ambion.com), as per the manufacturers' instructions. Universal human RNA (Stratagene, http://www.stratagene.com) was amplified in the same way. Probes for microarray hybridization were prepared by labeling 3 μg of amplified RNA with Cy-3 (universal RNA; green values) or Cy-5 (CD4^+^ or CD8^+^ T cells; red values) by reverse transcription. Detailed information on the microarrays as well as the labeling and hybridization procedures can be obtained at The Microarray Centre of The Toronto University Health Network (http://www.microarrays.ca/).

### Microarray Data Preprocessing

Microarrays were scanned at 16 bits using the ScanArray Express scanner (Packard Bioscience, http://las.perkinelmer.com) at 10-μm resolution at 635 (red)- and 532 (green)-nm wavelengths for Cy-5 and Cy-3, respectively, to produce image (tiff) files that were quantified using Genepix Pro 6.0 image-analysis software (Molecular Devices Corporation, http://www.moleculardevices.com). Bad spots were flagged manually according to their morphologies. The results were saved as Quantarray files where the intensity values ranged from 0 to 2^16^ − 1 (65,535) units. The tiff and Quantarray files were compressed and archived for permanent storage and further analysis. The microarrays were then screened for quality, first by visual inspection of the array with flagging of poor-quality spots, and second with automated scripts that scanned the quantified output files and measured overall density distribution on each channel and number of flagged spots. Box plots and density-distribution plots were drawn and inspected. Each quantified output file was run through the following preprocessing steps using the R language and environment (http://www.r-project.org) and the Limma package [25]. For minimum-intensity filtering, red and green values were treated with a surrogate-value replacement policy for estimating subthreshold values. For normalization within arrays, the raw merged red and green channels were lowess-normalized (grouped by print tip) and transformed to log_2_ ratios [26]. The commensurability of average brightness between the arrays of a pool of arrays was then assured using zero-centering of log-distributions normalization. For the ImmunArray design (The Microarray Centre of The Toronto University Health Network), each clone (gene) is represented by two independent spots, to provide for internal replicates. When both duplicate spots of a clone (gene) passed quality control, the average value of the duplicate clones was calculated and used as the representative value for that gene. If only one of the clone duplicate spots passed quality control, only that value was used in the downstream analysis. All data were then represented as log_10_ (red/green) expression ratios for further analysis.

### Quantitative Real-Time-PCR

Total RNA was reverse transcribed in a final volume of 50 μl using the High Capacity cDNA Archive kit with random primers (Applied Biosystems, http://www.appliedbiosystems.com) as described by the manufacturer. Reverse-transcribed samples were quantified using spectrophotometer measurements, diluted to a concentration of 5 ng/μl, and stored at −20 °C. A reference RNA (human reference total RNA [Stratagene]) was also transcribed to cDNA and was used as the calibrator. Gene-expression levels were determined using primer and probe sets from Applied Biosystems (ABI Assays on Demand [http://www.appliedbiosystems.com/]). The human *glyceraldehyde-3-phosphate dehydrogenase (GAPDH)* predeveloped TaqMan assay (PN4326317E) was used as the endogenous control. PCR reactions were performed using 4 μl of cDNA samples (20 ng), 5 μl of the TaqMan Universal PCR Master mix (Applied Biosystems), and 0.5 μl of the TaqMan Gene Expression assays (20×) in a total volume of 10 μl. The ABI PRISM 7900HT Sequence Detection system (Applied Biosystems) was used to detect the amplification level, and was programmed to an initial step of 10 min at 95 °C, followed by 40 cycles of 15 s at 95 °C, and 1 min at 60 °C. All reactions were run in triplicate, and the average values of the triplicates were used for quantification. The relative expression level of target genes was determined by using the ΔΔCT method. Briefly, the CT (threshold cycle) values of target genes were normalized to an endogenous control gene *(GAPDH)* (ΔCT = CT*_target_* − CT*_GAPDH_*) and compared with a calibrator (human reference RNA): ΔΔCT = ΔCT_sample_ − ΔCT_calibrator_. Relative expression (RQ) was calculated using the Sequence Detection system (SDS) 2.2.2 software (Applied Biosystems) and the formula RQ = 2^−ΔΔCT^.

### Student's t-Test and Linear Discriminant Analysis

The well-established univariate Student's t-test can determine whether the differences in expression for each gene are statistically significantly different in the aGVHD+ versus the aGVHD− and the cGVHD+ versus the cGVHD− sample classes, respectively. Specifically, given knowledge of the GVHD+ and GVHD− class arithmetic means and standard deviations from measurements, the t-test provides the probability or *p*-value of rejecting the null hypothesis of equal class means, given the null hypothesis being true (i.e., that both sample classes are essentially indistinguishable and derive from the same underlying distribution). It is also well established in practice that the t-test is robust against substantial departures from normality [27]. However, the t-test does not address per se the question of the robustness of class-prediction accuracy for a predictive model. A clinical user of such a model would ultimately like to predict whether a donor sample falls in the GVHD+ or GVHD− class, and what the expected accuracy and robustness of such a prediction would be. To this end, we used linear discriminant analysis (LDA) to estimate the accuracy of GVHD predictive genes discovered in microarray and quantitative real-time (qRT)-PCR experiments [28]. In addition, we assessed the robustness for all the genes validated by qRT-PCR by performing 500 independent instances of training-test dataset splits cross-validation to determine empirically through computational resampling the expected generalizable class-prediction accuracy on independent test datasets [29,30]. In LDA with assumed equal class a priori probabilities, the boundary between class P (GVHD+) and class N (GVHD−) is determined by the value of the separatrix, S, which is the point (in univariate analysis) between the class P and N means that is equidistant to both [28]. If the observed mean of class P is smaller than the observed mean of class N, all values less than or equal to S will be classified by the model as P, and all values greater than S will be classified as N. When the observed mean of class P is greater than the observed mean of class N, all values greater or equal to S will be classified by the model as P, and all values smaller than S will be classified as N. For all the samples that were classified by the model as P, the ones that also correspond to known P samples in the measured data constitute true positives, and the ones that correspond to known N samples in the measured data constitute false positives. For all the samples that were classified by the model as N, the ones that also correspond to known N samples in the measured data constitute true negatives, and the ones that correspond to known P samples in the measured data constitute false negatives. Accuracy rate is (true positives + true negatives)/total number of samples. Sensitivity is true positives/(true positives + false negatives), and specificity denotes true negatives/(true negatives + false positives).

### Predictive Interaction Analysis

Predictive interaction analysis (PIA) was carried out on the 105 gene pairs formed by 15 genes that were individually predictive of GVHD in both microarray and qRT-PCR experiments. Gene pairs and single genes were compared as to their ability to distinguish GVHD+ from GVHD− samples according to the statistical methods outlined below.

#### Two-class discriminant analysis.

Standard equations of LDA [28] are employed for determining two-class separations (GVHD+ versus GVHD−), based on single-gene or two-gene abundances. Column vector 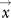 represents the log_10_ abundances of a gene pair (mapping of gene pair abundances to a single variable is defined in PIA below). *T* denotes transpose. The variable *c*1 denotes one known class (e.g., GVHD+), and *c*2 denotes a second known class (e.g., GVHD−). The general two-class linear discriminant equation [28] is:

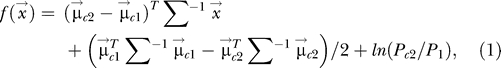
where gene pair vectors 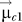 and 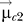 are the respective class means; Σ^−1^ is the inverse of the gene pair by gene-pair data-derived pooled covariance matrix Σ, which is the sample number-weighted sum of the data-derived within-class covariance matrices. *P_c_*_1_ and *P_c_*_2_ are the prior probabilities of the two classes. The *ln(P_c_*_2_ */P_c_*_1_ *)* term in Equation 1 is zero because we set *P_c_*_2_ = *P_c_*_1_. In the LDA we are performing, the proportion of c2 samples compared to c1 samples in the data is not germane. Of relevance in the LDA are the individual sample data values, the class means, and the within-class variations, not the class prior probabilities per se*.* Setting Equation 1 to zero defines the general equation for the separatrix L:

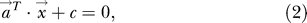
where parameter vector 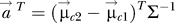 and scalar 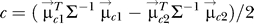 are data-dependent constants. The general L then can be written immediately in slope/intercept form as

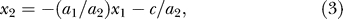
where 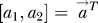 . However, in the PIA to be described below we use a specialized, deliberately constrained form of Equation 3. Namely, the separatrix L has slope −1 (synergistic PIA [SPIA]), or +1 (competitive PIA [CPIA]), and necessarily bisects the chord between the two class means 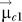 and 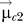 .

#### CPIA and SPIA.

The product *X* × *Y* for gene *X* and gene *Y* represents a synergistic phenomenological gene–gene interaction (SPIA), and the abundance ratio *X/Y* (or *Y/X*) for gene *X* and gene *Y* represents a competitive phenomenological gene–gene interaction (CPIA). We define *x* = *log*_10_ *(X), y* = *log*_10_ *(Y),* and new coordinates or axes: *u* = *x* + *y* and *v* = *x* − *y*. Class separation in *(x, y)* with respect to univariate *u* is termed SPIA, and class separation with respect to univariate *v* is termed CPIA. PIA refers to either SPIA or CPIA. Hence, good class separation in SPIA is demonstrated by good separation in *(x, y)* by a separatrix *u* = *x* + *y* = *constant* (equivalent to *y* = −*x* + *constant,* i.e., slope −1), and good class separation in CPIA is demonstrated by good separation in *(x, y)* by a separatrix *v* = *x* − *y* = *constant* (equivalent to *y* = *x* − *constant*, i.e., slope +1). Thus, we apply LDA under models restricted to separatrices whose slopes are constrained deliberately to −1 or +1.

#### Classification performance measures.

We use straightforward sampling statistics to characterize class separation by *p*-values, as well as by counts of correctly classified samples relative to the total number of samples being classified (univariate LDA accuracies). The class-separation performance of a gene pair *(X,Y)* in SPIA or CPIA can be assessed readily on single axes *x, y, u,* and *v*. When samples in *(x,y)* are, for example, projected onto the *x*-axis, classification performance is assessed by the *p*-value returned by a simple homoscedastic t-test for differences of two means. This is computed analogously and separately for the *y*-, *u*-, and *v*-axes. It is important—because of the biological interpretations offered by SPIA and CPIA—to focus on those gene pairs for which two-class separation (as assessed by intercomparable *p*-values) is better in *u* or in *v* than in *x* and in *y*. Thus, we seek gene pairs *(X,Y)* for which along the “single variable” *u*-axis or *v*-axis, the classes separate better than along the *x*-axis only and better than along the *y*-axis only.

## Results

### Experimental Model

**Figure 1:**
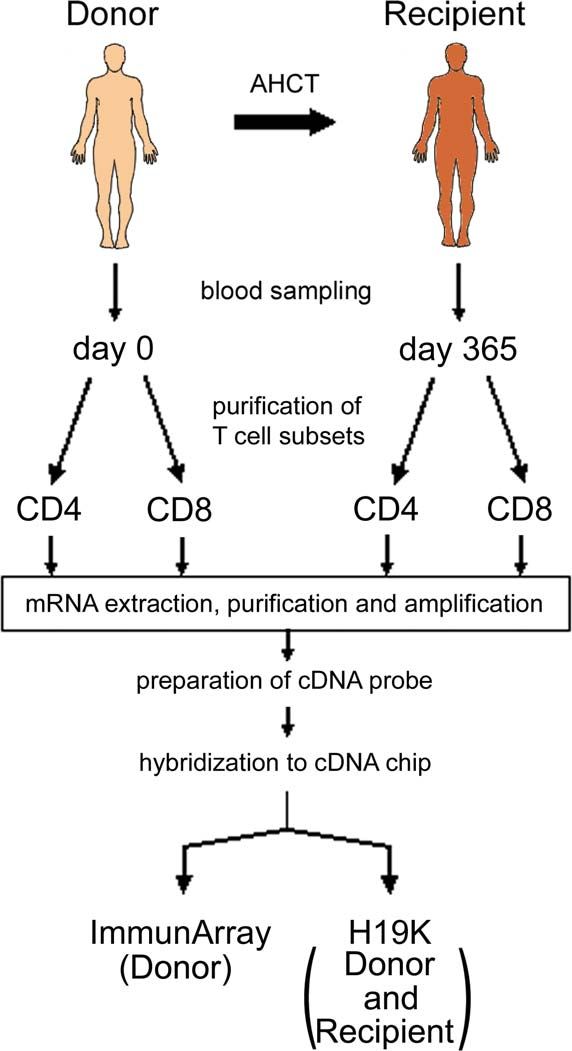
Study Design Donor and recipient T cells were obtained on days 0 and 365, respectively. Total RNA from purified CD4^+^ and CD8^+^ T cells was reversed transcribed and hybridized on the human H19K array (donor and recipient T cells) and the ImmunArray (donor T cells) from The Microarray Centre of The Toronto University Health Network.

In our quest for a GVHD-predictive signature, our prime objective was to correlate global gene-expression profiling of AHCT donor T cells with the occurrence of GVHD in recipients. A secondary objective was to evaluate whether the donor gene-expression profile persisted long-term in the recipient. Peripheral blood was obtained from 50 AHCT donors pretransplant (referred to as day 0) and from 40 recipients on day 365 (ten recipients were dead by day 365) (Figure 1). Donors and recipients were human leukocyte antigen-identical siblings. Recipients were regarded as negative for aGVHD when they lived at least 100 days without presenting GVHD. Recipients were considered negative for cGVHD when they remained cGVHD-free for 365 days post-AHCT. CD4^+^ and CD8^+^ T cell subsets were purified with microbeads. Total RNA was purified, amplified, reverse transcribed, and hybridized on microarrays developed by The Microarray Centre of The Toronto University Health Network. RNA from donor and recipient T cells was hybridized on the human H19K array (19,008 expressed sequence tags), and donor T cell RNA was also hybridized on the ImmunArray (3,411 ESTs from immune-related genes). The ImmunArray provides additional genes for better coverage of immune responses to complement the H19K array.

The success rate of gene-expression profiling studies decreases with the degree of biological noise inherent to the experimental system [31–34]. Accordingly, our study design included four features to reduce biological noise. First, unlike recipients of solid organ grafts who inevitably present organ failure (e.g., renal insufficiency), AHCT donors are healthy individuals. This is important because serious ailments (and their treatment) cause alterations in global gene expression that are significantly greater than the background variation in normal gene expression [35]. Second, our studies were performed on purified CD4^+^ and CD8^+^ T cells because cell lineage is a primary determinant of gene-expression profile [36], and the transcriptome of CD4^+^ and CD8^+^ T cells shows significant differences [37]. Third, CD4^+^ and CD8^+^ T cells are necessary and sufficient for induction of antiminor histocompatibility antigen GVHD [38,39], the clinical endpoint of this study. Fourth, AHCT recipients were treated in a single center using standardized therapeutic regimens and uniform criteria for diagnosis of GVHD.

### Donor T Cell Gene-Expression Profiling Using Microarrays

**Figure 2:**
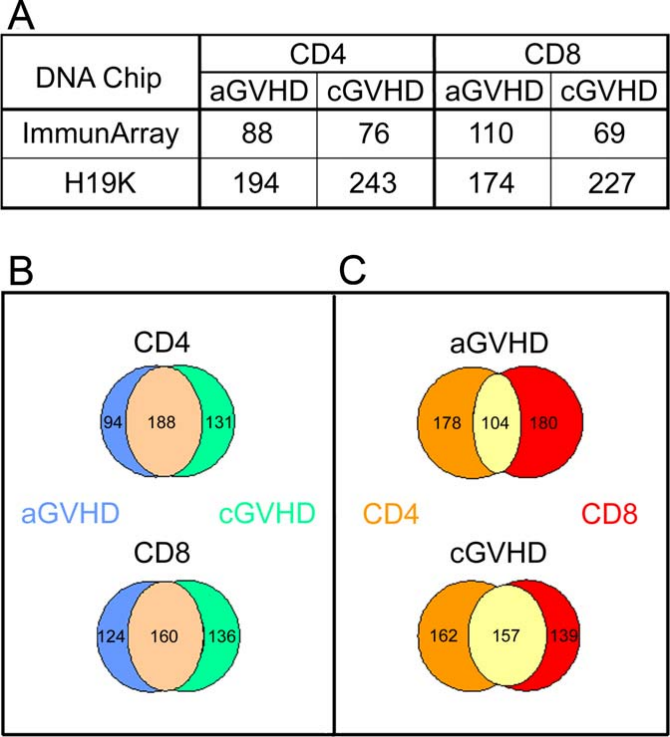
GVHD Predictive Genes Identified by One-Dimensional Analyses Searches were performed using two methods: a linear discriminant-analysis–based approach and statistical t-test. (A) Number of genes showing a GVHD-predictive accuracy ≥ 65% and *p* ≤ 0.05. (B and C) Data from the H19K and ImmuArray were pooled. Among GVHD-predictive genes, Venn diagrams represent counts relationships between CD4^+^ versus CD8^+^ T-cell gene profiles (B) and aGVHD versus cGVHD predictive-genes (C).

We first carried out eight searches for class-discriminating genes using two methods, a statistical t-test and a specially constrained LDA, over four class divisions. Class divisions were, for CD4^+^ and CD8^+^ T cells: (i) recipients with no GVHD versus those with aGVHD (with or without cGVHD); and (ii) recipients with no GVHD versus those with cGVHD (with or without aGVHD). Recipients were considered GVHD− only when they presented no signs of GVHD after a minimum follow-up of one year post-AHCT. We selected for analysis genes showing a GVHD-predictive LDA accuracy (ability to discriminate donors whose recipient presented GVHD or not) ≥ 65% and class discrimination t-test *p* ≤ 0.05 (Figure 2A). Consistent with the notion that aGVHD strongly correlates with cGVHD [19], many of the genes predictive for aGVHD were also predictive for cGVHD (Figure 2B). A substantial proportion of GVHD-predictive genes were common to both CD4^+^ and CD8^+^ donor T cells (Figure 2C). However, the fact that most GVHD-associated genes were found in only CD4^+^ or CD8^+^ T cells supports the need to analyze T cell subsets independently (Figure 2C). Among genes emerging from the ImmunArray and H19K datasets, those that are annotated and have a demonstrated or putative function in T cell biology are listed in Table S1 (genes overexpressed in GVHD+ relative to GVHD− donors) and Table S2 (genes repressed in GVHD+ donors). Overall, the numbers of genes that were up-regulated/down-regulated in GVHD+ relative to GVHD− donors were 22/42 for CD4^+^ T cells and 31/40 for CD8^+^ T cells. About 60% of these genes are involved in cell proliferation, signal transduction, or transcription (unpublished data).

### qRT-PCR Analyses of GVHD-Predictive Genes

#### Predictive value of single genes.

To evaluate the validity of predictive genes identified with microarrays, we performed qRT-PCR analyses on fresh mRNA aliquots extracted from donor CD4^+^ (*n* = 33) and CD8^+^ (*n* = 35) T cells. We focused on cGVHD-predictive genes and tested a total of 26 genes, including 24 genes present in Table S1 and Table S2. We selected the latter 24 genes based on two criteria: they are involved in cell proliferation and/or cytokine signaling and were differentially expressed in cGVHD+ versus cGVHD− donors. Analyzing several genes involved in a common signaling cascade has special interest because it provides a unique opportunity to validate the biological coherence of differentially expressed genes. Preliminary analysis of Table S1 and Table S2 showed that at least five cGVHD-predictive genes were components of the transforming growth factor-β (TGF-β) signaling pathway. These five genes were selected for quantitative PCR studies. To further evaluate the possible role of the TGF-β pathway, we also tested *TGIF* and TGF-β-induced *(TGFBI)* (which were not present on the microarrays), which are transcriptional targets of TGF-β. Performance of individual genes was evaluated using univariate Student's t-test and LDA. The statistical significance corresponds to t-test *p*-value, whereas classification performance (sensitivity, specificity, and overall accuracy) was derived from LDA.

**Figure 3:**
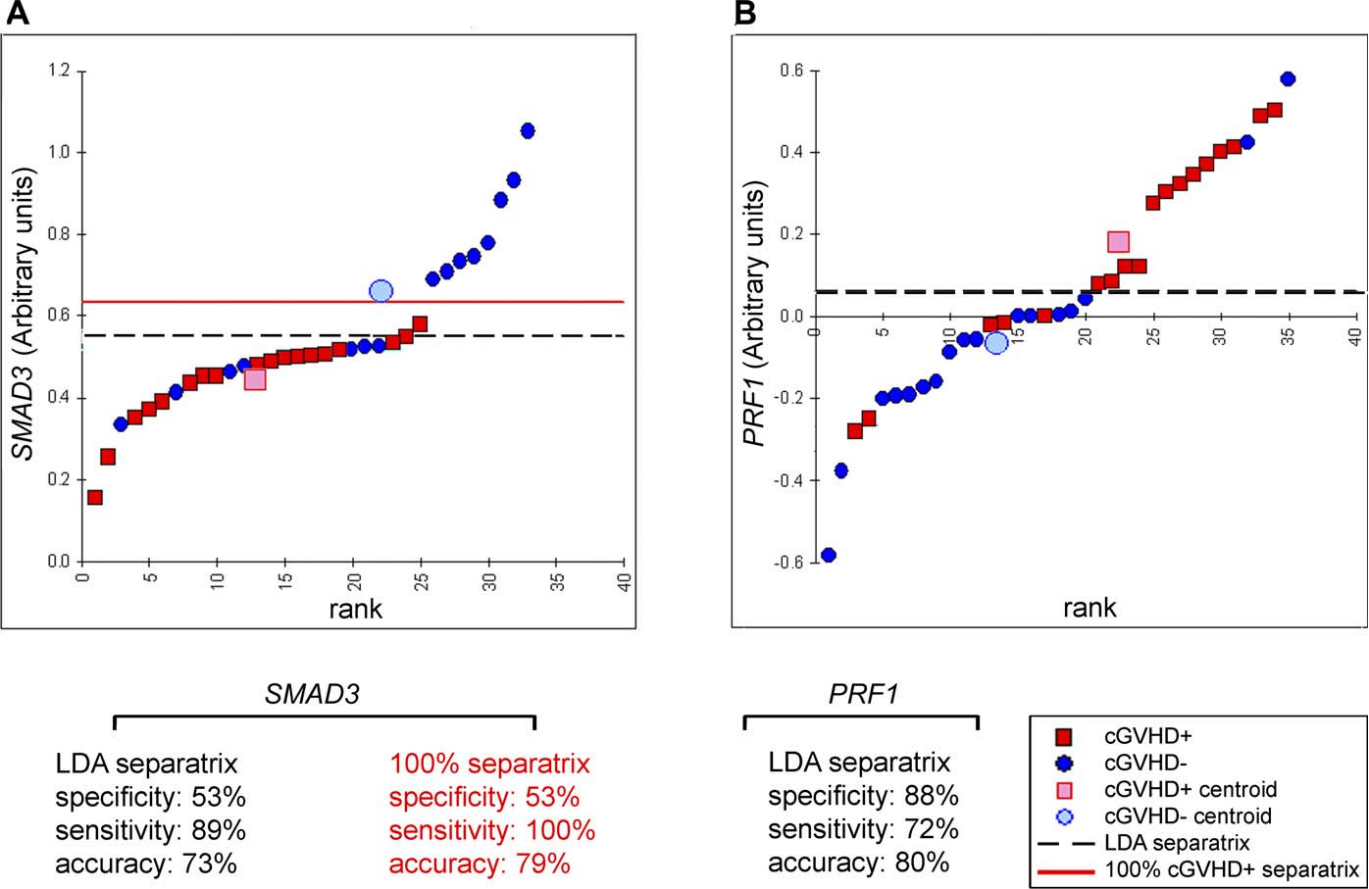
LDA-Based Scatterplot of qRT-PCR Data for *SMAD3* and *PRF1* Levels of (A) *SMAD3* and (B) *PRF1* transcripts were assessed in CD4^+^ and CD8^+^ T cells, respectively. Data for all donors tested by qRT-PCR were ranked according to relative gene expression levels. Thick horizontal black line corresponds to the LDA separatrix. For *SMAD3,* a computationally repositioned separatrix for 100% GVHD+ discrimination is shown (red line).

qRT-PCR did not confirm the predictive value of nine genes (Table 2). This result can be explained by the limited sample size and the idiosyncrasies of the two mRNA-measurement procedures (e.g., cross-hybridization and splicing variants) [34]. Out of the 26 genes tested, 17 were differentially expressed in GVHD+ and GVHD− donors (Table 2): 15 genes selected from Table S1 and Table S2 (they showed consistent change directionality in microarrays and qRT-PCR) plus the two supplementary TGF-β target genes. The statistical significance (t-test *p*- value) of individual cGVHD-predictive genes ranged from 0.046 to 0.0005, and their GVHD-predictive accuracy (LDA) from 63% to 80%. Of note, there was a weak negative correlation (*r* = −0.53, *p* = 0.03) between the specificity and sensitivity of the 17 genes. Thus, some genes were better in predicting the occurrence of GVHD than its absence, and vice versa for other genes. *PRF1* showed the best specificity (Figure 3; Table 2). *PRF1* codes for perforin, whose high expression in CD8^+^ T cells was associated with occurrence of GVHD. *SMAD3,* a transcription factor that is activated following TGF-β binding, showed the highest sensitivity (Figure 3; Table 2). High levels of *SMAD3* transcripts in CD4^+^ T cells correlated with absence of GVHD. Based on the LDA-generated class separatrix, the specificity and sensitivity for *SMAD3* were 53% and 89% with an overall accuracy of 73%. We repositioned post-hoc the separatrix in order to have all cGVHD+ donors on one side of the separatrix (hereafter referred to as the 100% cGVHD+ separatrix). This new separatrix, which by definition increased the sensitivity to 100%, also increased the overall accuracy to 79% without changing the specificity (Figure 3). Thus, low levels of *SMAD3* were found in all GVHD+ and some GVHD− donors, while all donors expressing high levels of *SMAD3* were GVHD− (Figure 3). Mechanistically, this suggests that high levels of *SMAD3* are sufficient (but not necessary) to prevent GVHD, while low levels are necessary (but not sufficient) for the occurrence of GVHD.

**Table 2:**
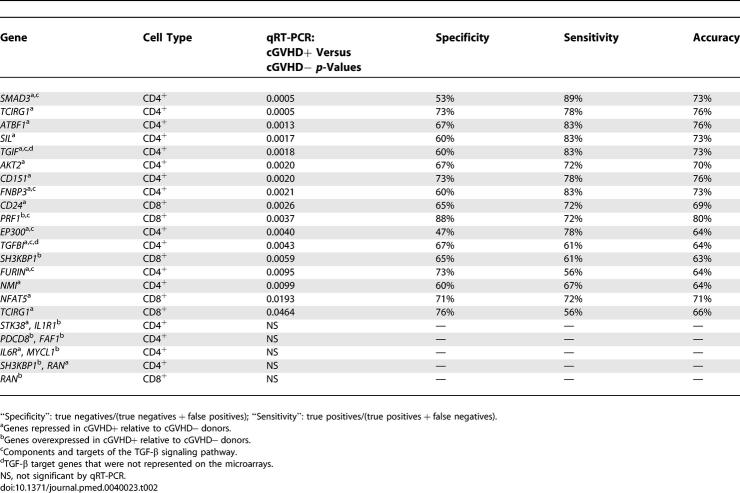
qRT-PCR Analyses of GVHD-Predictive Genes

One major point highlighted by gene-expression profiling studies is the primacy of pathways over the effects of individual genes (pathways ultimately define the profiles) [36,40]. With this in mind, a most salient finding was that all components and targets of the TGF-β pathway tested by qRT-PCR were differentially expressed in GVHD+ versus GVHD− donors (Table 2). Compared with GVHD+ donors, GVHD− donors showed up-regulation of *EP300, FURIN, FNBP3, SMAD3, TGFBI,* and *TGIF,* and repression of *PRF1.* From a pathway perspective, that expression profile is entirely consistent and points to increased TGF-β signaling in T cells from GVHD− relative to GVHD+ donors [41–47]. The ten other cGVHD-predictive genes whose differential expression was confirmed by qRT-PCR are involved in regulation of cell growth and proliferation *(AKT2, ATBF1, CD24, CD151, MYCL1, NFAT5*, *NMI, SIL, SH3KBP1,* and *TCIRG1*) [48–57].

#### PIAs using a pairwise interaction model.

**Figure 4:**
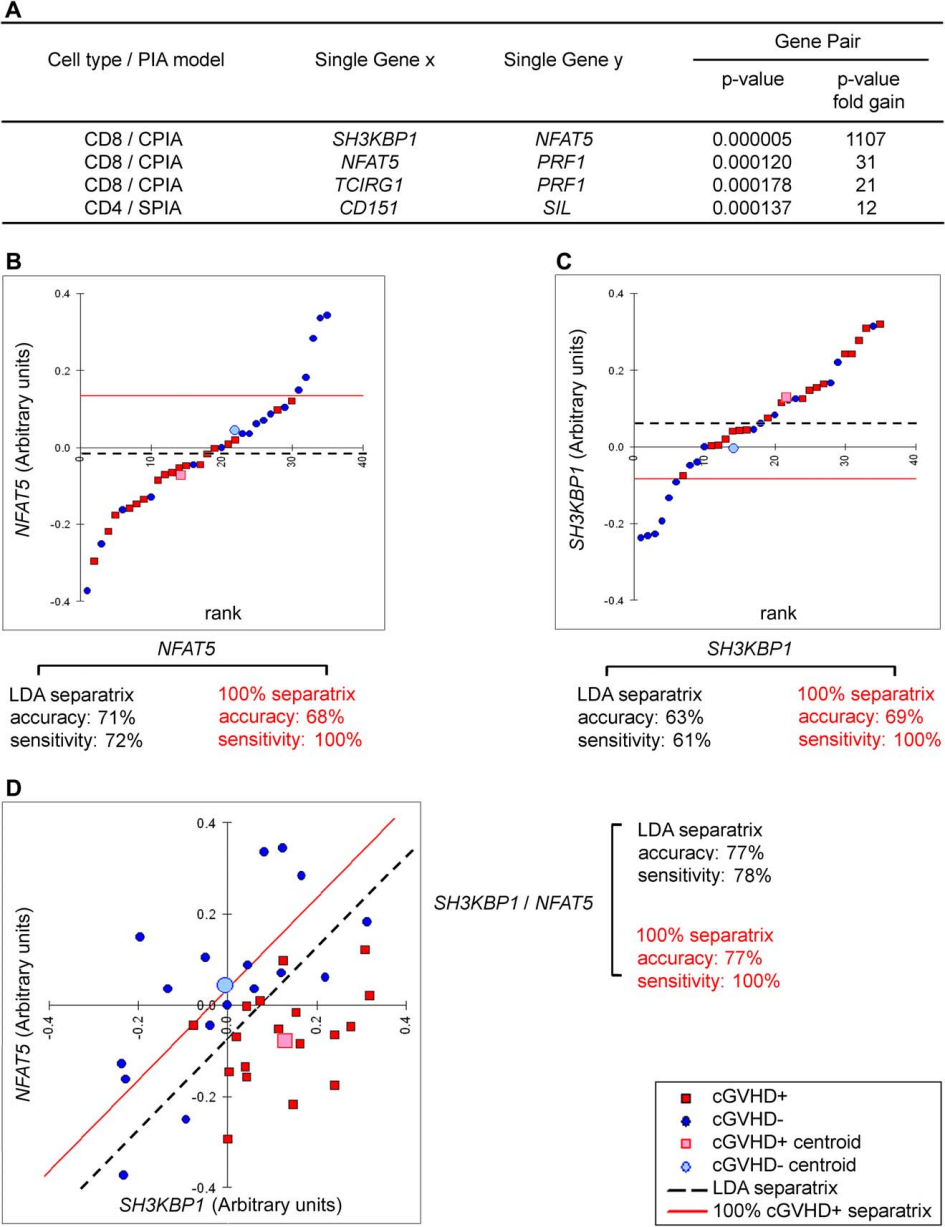
Competitive and Synergistic Interactions between GVHD-Predictive Genes (A) PIA identified four gene pairs whose *p*-value for cGVHD prediction was at least 10-fold lower than that of constituent genes. LDA-based scatterplots of qRT-PCR data for (B) *NFAT5,* (C) *SH3KBP1,* and (D) the *NFAT5/SH3KBP1* gene pair. Dotted lines represent LDA-generated separatrices. Red lines correspond to 100% cGVHD+ separatrices (designed to maximize sensitivity).

A global approach is required to properly understand cellular responses, because interpathway cross-talk and other properties of networks reflect underlying complexities that cannot be explained by the consideration of individual pathways in isolation [58,59]. In their simplest form, gene–gene interactions may be phenomenologically competitive or synergistic. We posited that such interactions might be reflected in particular gene-pair expression patterns. For example, if gene *X* and gene *Y* represent a competitive interaction, the ratio of gene *Y/X* expression should determine GVHD outcome (e.g., presence and absence of GVHD will correlate with high and low *Y/X* ratios, respectively). Alternatively, for synergistic interactions, the occurrence of GVHD should be regulated by the product of genes' *X* × *Y* activities. We therefore examined gene-pair expression ratios and products within the context of competitive and synergistic models. To this end, we evaluated the gene pairs formed by the 15 GVHD-predictive genes validated in both microarray and qRT-PCR experiments (Table 2). The total number of gene pairs analyzed corresponds to *n*(*n* − 1)/2 (i.e., 105). We asked whether CPIA and SPIA would highlight gene pairs whose *p*-value for cGVHD versus no GVHD was at least 10-fold lower than that of constituent genes. A total of four gene pairs satisfied this stringent criterion (Figure 4A). PIAs suggest that *NFAT5,* a transcription factor that regulates gene expression induced by osmotic stress [53], has competitive interactions with *SH3KBP1* (alias *CIN85*), which interacts with CBL (a negative regulator of immune signaling) [56], and with *PRF1,* a quintessential component of CD8^+^ T cell granule exocytosis cytotoxicity pathway [60]. Likewise, PIAs suggest that *PRF1* has competitive interactions with *TCIRG1* (alias *TIRC7*), a negative regulator of T cell activation and cytokine response [57]; and that *CD151,* a negative regulator of Ag-induced T cell proliferation [51], collaborates synergistically with *SIL,* a gene whose expression is associated with cell proliferation [61]. From a mechanistic perspective, these data suggest that interactions between the four pairs' constituent genes are biologically relevant and should be investigated.

Gene pairs discovered by PIA can provide better performance than constituent single genes in terms of prediction accuracy. Performance gain is illustrated by further analyses of the *SH3KBP1/NFAT5* gene pair using LDA and two class-separatrices: the LDA-generated separatrix and the 100% cGVHD+ separatrix (designed to maximize sensitivity) (Figure 4). Compared to the LDA-generated separatrix, the 100% cGVHD+ separatrix increased the sensitivity by 22%–39% without compromising overall accuracy (Figure 4). Using the LDA-generated separatrix, the *SH3KBP1/NFAT5* gene pair provided a 6% gain in both sensitivity and overall accuracy compared with single genes. With the 100% cGVHD+ separatrix (which by definition gives a 100% sensitivity), the overall accuracy gain was 8%. From a clinical standpoint, these data suggest that PIAs can identify gene pairs with greatly enhanced predictive accuracies and stronger *p*-values compared to their constituent genes. Furthermore, they imply that in further studies with a larger number of participants, higher-order combinatorial searches could significantly improve the prediction performance of gene-expression profiling [30].

#### Multiple training-test dataset split cross-validation.

We can be confident that genes with good cGVHD+- and cGVHD−-differentiating t-test *p*-values over the complete set of samples have a statistically significant ability to distinguish between these classes (in terms of rejecting the equal means null hypothesis). However, the assessment of LDA classification accuracy on a single set of samples may not be robust, since accuracy could be highly sensitive to chance fluctuations of measurement points in the vicinity of the separatrix. Such situations might not have a large impact on *p*-value, but can disproportionately affect accuracy assessments. To establish whether cGVHD+/− discrimination accuracy may be generalizable and robust, we need to determine the accuracy of the model prediction on test datasets that are independent (with regard to sampling) of the training datasets from which the predictive LDA models are derived. However, a single instance of training-test dataset comparison can be considered neither representative nor robust, since it is potentially sensitive to idiosyncratic fluctuations of datapoints around the separatrix. We therefore determined the robust average accuracy over many independently generated test datasets for each gene, on the basis of different selections of training-set data for each gene [30], using conventional cross-validation procedures [29]. These analyses were performed on the 17 single genes (Table 2) and the PIA variables representative of the four gene pairs (Figure 4A) that were predictive of cGVHD occurrence. Specifically, for each gene, we carried out 500 different 60% training samples and 40% test-samples dataset splits by randomly assigning (for each data split) 60% of the respective cGVHD+ and cGVHD− samples to a training dataset, and the remaining 40% of the samples to the respective test datasets. For CD4^+^ cells, 11 cGVHD+ and nine cGVHD− samples were selected randomly for training datasets, while the seven cGVHD+ and six cGVHD− remaining samples were used in test datasets. For CD8^+^ cells, 11 cGVHD+ and ten cGVHD− samples were selected randomly for training datasets, while the remaining seven cGVHD+ and seven cGVHD− samples were used in test datasets. The test dataset accuracy was determined separately for each of the 500 training/test random-sampling splits by using the LDA-predictive model separatrix from the corresponding training dataset. We emphasize that each test dataset-accuracy determination for each gene was carried out 500 separate times on randomly chosen dataset splits, each time using a predictive model that has never been exposed to the test data.

**Figure 5:**
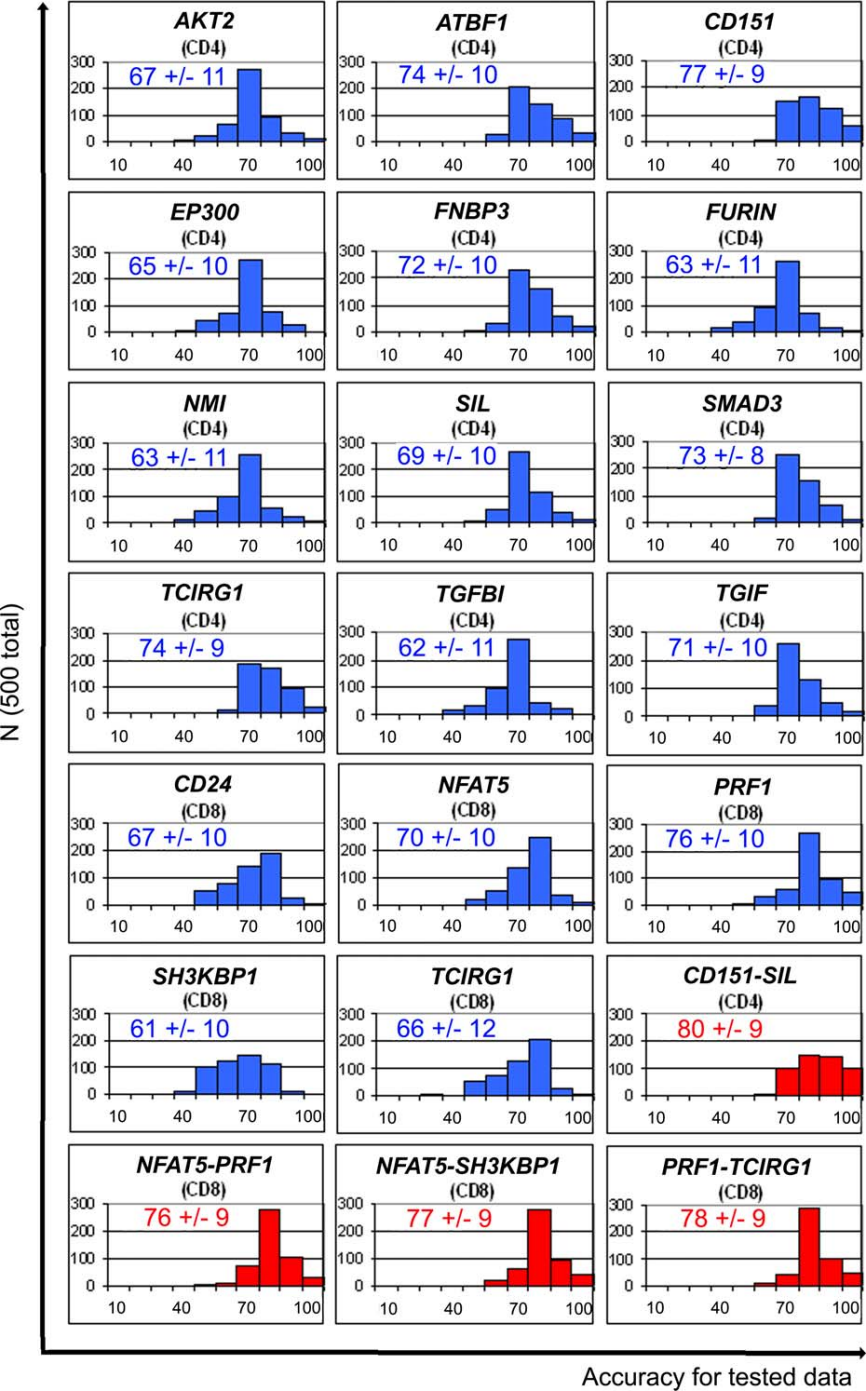
Multiple Training-Test Dataset Split Cross-Validation For each single gene (*n* = 17, blue) and gene pairs (*n* = 4, red), we carried out 500 different 60% training samples and 40% test samples dataset splits by randomly assigning 60% of the respective cGVHD+ and cGVHD− samples to a training dataset and the remaining 40% of the samples to the respective test datasets. The test dataset accuracy was determined separately for each of the 500 training/test random sampling splits by using the LDA predictive model separatrix from the corresponding training dataset. Bar graphs show the occurrence of specific accuracies in 10% accuracy increments. Numbers in each panel represent the mean test-set accuracy (%) ± standard deviation.

We report for each gene the robust cross-validation ensemble average test-set accuracy and its standard deviation, as well as bar graphs depicting occurrences of specific accuracies in 10% accuracy increments (Figure 5). We found that the average test-set cross-validation accuracy was 71% ± 10%, and that genes such as *CD151* for CD4^+^ cells achieved an accuracy of 77% ± 9%, and *PRF1* for CD8^+^ cells achieved 76% ± 10%. Notably, the test-set cross-validation accuracy of gene pairs identified by PIA often outperforms that of single genes. For example, the *CD151–SIL* gene pair achieved 80% ± 9%, while its constituent genes *CD151* and *SIL* provided accuracies of 77% ± 9% and 69% ± 10%, respectively. In addition, in Figure 5 we see a conspicuous shift of occurrences of accuracies from the 70% and 80% histogram bins for the constituent genes to the 90% and 100% bins for the gene pairs. These data provide strong evidence that the 17 genes and four gene pairs reported herein not only show statistically significant differences between cGVHD+ and cGVHD− donors, but also that these differences are substantial in magnitude and robustly provide higher than 70% accuracies overall. We therefore infer that the robust discrimination performance of these genes and gene pairs could be of clinical value for cGVHD prediction.

### The Microarray-Based Donor Gene Profile Persists Long-Term in the Recipient

To determine whether differences in donor gene-expression profiles were transferable, we evaluated whether they persisted in the recipient. All our recipients were adults that were given a myeloablative-conditioning regimen and received a non-T cell–depleted AHCT. In these conditions, essentially all T cells on day 365 are donor-derived [62–65]. We therefore studied the relationship between the donor gene profiles on day 0 (*t*0) and the recipient profiles on day 365 (*t*3). In other words, we compared the transcriptome of T cells derived from a single zygote (the donor) but residing in two types of environments (the donor and the recipient). To get a manageable yet broad basis for analyses, we included two gene sets tested on the H19K chip: the top 400 genes showing differential expression in GVHD+ versus GVHD− donors on day 0, combined with the top 400 genes showing differential expression in GVHD+ versus GVHD− recipients on day 365 (Table S3). Because of overlap between the two gene sets, a total of 711 genes was analyzed. Genes that exhibited little variation across arrays were excluded because they do not contribute useful information for distinguishing among specimens [36]. The basic postulate underlying our analyses was that if the donor profile is largely transferred to the recipient, correlation between a donor on day 0 and its recipient on day 365 (*t*0i − *t*3i) would be stronger than (i) correlation of that donor with other donors on day 0 (*t*0i − *t*0) and (ii) correlation of that recipient with other recipients on day 365 (*t*3i − *t*3). The reverse would be true, and the donor-specific characteristics should be “washed out,” if the gene-expression profiles were either unstable or regulated primarily by adaptive (environmental) effects.

**Figure 6:**
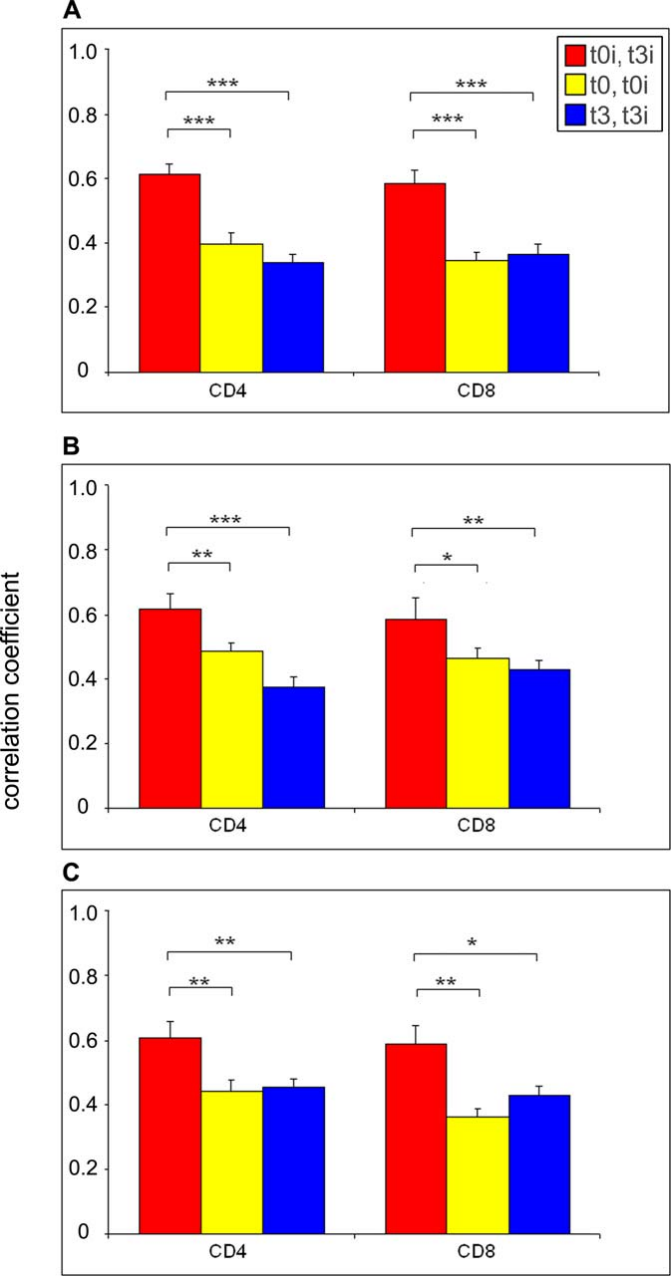
The Pre-AHCT Donor Gene Expression Profile Correlates with the Recipient Expression Profile Examined One Year Post-AHCT The Pearson correlation coefficient (rho) over the expression vectors of 711 informative genes (listed in Table S3) was calculated between members of all matching donor–recipient pairs, and all donor–donor and recipient–recipient pairs, and then averaged for each group. Bar graphs show the mean Pearson correlation coefficient between individual donors on day 0 with their recipient on day 365 (*t*0i − *t*3i) (red bar), between individual donors and all other donors on day 0 (*t*0i − *t*0) (yellow bar), and between individual recipients and all other recipients on day 365 (*t*3i − t3) (blue bar). Data are from all (40) donor–recipient pairs (A), or from pairs in which the recipient presented cGVHD (B), or not (C). Error bars represent the standard error of the mean. The vector of (*t*0i − *t*3i) correlations was compared to the vectors of (*t*0i − *t*0) and (*t*3i − *t*3) correlations using Student's t-test, to determine whether the differences between these observed sample pair correlation groups are statistically significant. t-Test *p*-values relative to (*t*0i − *t*3i) are labeled as follows: *, 0.01 < *p* < 0.05; **, 0.001 < *p* < 0.01; ***, *p* < 10^−6^.

We found that the average gene-expression profile correlation among corresponding donor–recipient pairs (*t*0i − *t*3i) was consistently higher than the average correlation among donors (*t*0i − *t*0) and among recipients (*t*3i − *t*3) (Figure 6). This was true for CD4^+^ and CD8^+^ T cells, in recipients that were cGVHD+ and those that were cGVHD− (Figure 6). Thus, interindividual differences in expression of GVHD-associated transcripts are remarkably stable over time (365 days). Stability over time increases their potential value as predictive markers. The donor gene-expression profiles are also very robust since they persist following transfer in a different host (the recipient) even in the presence of confounding disease-related factors (cGVHD and its treatment). The stability and “transferability” of the GVHD-linked gene-expression profiles point to a major genetic (as opposed to environmental) influence. Since donors and recipients were siblings it is formally possible that the similar environments (nonhematolymphoid cells) in which T cells resided may have contributed to the transferability of the T cell-expression profiles.

## Discussion

Several conclusions can be drawn from our work. First, the donor gene-expression profile has a dominant influence on the occurrence of aGVHD and cGVHD in the recipient. Second, extensive studies on cGVHD prediction revealed that the “dangerous donor” trait (occurrence of GVHD in the recipient) is under polygenic control and is determined by competitive and synergistic gene interactions. Third, the risk of cGVHD is shaped by the activity of genes that regulate diverse cell functions in donor T cells, including TGF-β signaling and cell proliferation. Finally, the donor gene profile persists long-term in the recipient. We wish to emphasize that several convergent pieces of evidence underpin the robustness of conclusions presented herein: (i) in microarray experiments, the donor gene profile defined on day 0 showed exceedingly strong correlation with that of recipient CD4^+^ and CD8^+^ T cells harvested on day 365; (ii) for most genes tested by qRT-PCR, differential gene expression between cGVHD+ and cGVHD− donors was confirmed to be robust, on the basis of statistical tests and computational analysis of independent training-test datasets; (iii) from a pathway perspective, differential expression of TGF-β-related transcripts was entirely consistent with increased TGF-β signaling in T cells from cGVHD− relative to cGVHD+ donors. Compared with cGVHD+ donors, cGVHD− donors showed higher levels of activating components of the TGF-β signaling pathway *(EP300, FNBP3, FURIN, SMAD3)* and of genes induced by TGF-β *(TGFBI, TGIF)* but lower expression of *PRF1,* which is repressed by TGF-β (Table 2). Notably, transcripts for TGF-β *(TGFB1)* and its receptors *(TGFBR2* and *TGFBR3)* were represented on the microarrays and were not differentially expressed in T cells from cGVHD+ relative to cGVHD− donors (unpublished data). Collectively, these data suggest that under basal conditions interindividual variations exist in TGF-β signaling activity. Moreover, they imply that these interindividual variations are stable over time (Figure 6) and are due, at least in part, to differential expression of intracellular TGF-β pathway components rather than membrane-associated factors. The latter idea is consistent with recent data on Wnt and TGF-β signaling. Among thymocyte subsets, differential responsiveness to Wnt signals is not determined by expression of membrane-associated factors, but rather by the balance between activating and inhibiting intracellular components of the Wnt pathway (e.g., β-catenin, γ-catenin, and TCF-1) [66]. In addition, two recent studies demonstrated that modulation of SMAD proteins such as SMAD3 was sufficient to regulate the strength of TGF-β signaling [67,68].

To the best of our knowledge, our study is the first to present evidence that differential gene expression in donor CD4^+^ and CD8^+^ T cells is predictive of the risk of GVHD in the recipient. As mentioned in the Introduction, histoincompatibility is necessary but not sufficient to elicit GVHD. On the basis of our data, we propose that the occurrence of GVHD is determined by another key factor: a dangerous donor (strong alloresponder). Further studies are required to decipher how this complex polygenic trait is regulated. Nevertheless, the concept that TGF-β signaling in donor cells has a protective role against GVHD is consistent with the well-known pivotal function of TGF-β in maintaining tolerance and preventing the development of immunopathology [42]. TGF-β is the cytokine expressed constitutively at highest levels in lymphoid and nonlymphoid organs [69], and its pervasive influence on immune responses results from pleiotropic effects. TGF-β blocks T cell proliferation, inhibits differentiation of Th1 (T helper class 1) cells and CTLs (cytotoxic T lymphocytes), and promotes expansion as well as maintenance of CD4^+^CD25^+^ regulatory T cells that can inhibit GVHD [42,70–77]. Moreover, recent studies in mice have shown that production of TGF-β by donor T cells early after AHCT attenuates GVHD, and that neutralization of TGF-β significantly increases the severity of GVHD [78]. Since AHCT is generally used to treat hematologic malignancies, the fact that TGF-β has a tumor suppressor role in hematologic malignancies [79] might constitute an additional benefit associated with induction of the TGF-β pathway.

Among cGVHD-predictive genes that are not related to the TGF-β pathway, *TCIRG1* (alias *TIRC7*) is of particular interest, since it ranked first in terms of statistical significance for prediction of cGVHD (Table 2). GVHD− donors expressed higher levels of *TCIRG1* transcripts than GVHD+ donors. This is consistent with the function of TCIRG1, which colocalizes with the T cell receptor and mediates inhibitory signals that lead to up-regulation of CTLA4 and repression of interleukin-2 and interferon-γ [57,80]. Remarkably, TCIRG1-specific stimulatory antibodies significantly prolonged heart and kidney graft survival [81,82].

During the early months post-AHCT, recipient T cells derive to a large extent from proliferation of mature donor T cells present in the graft. However, by one year post-AHCT, recipient T cells derive mainly, if not exclusively, from development of donor-derived hematolymphoid progenitors in the recipient's thymus [83–85]. Thus, on day 365, recipient T cells originate essentially from donor hematopoietic stem cells as opposed to donor post-thymic T cells. The fact that the pre-AHCT donor gene profile correlates with the recipient profile one year post-AHCT (Figure 6) is therefore quite remarkable. These data provide compelling, albeit indirect, evidence that a significant portion of the differential gene profiles between GVHD+ and GVHD− donors is imprinted at the hematopoietic stem cell level. Moreover, stability of the gene-expression profiles in the donor and recipient over a one-year period suggests that the profiles result from inherited genetic traits as opposed to environmental factors. Genetic linkage analyses will be needed to test directly this inference.

Can identification of strong versus weak alloresponders be used to select AHCT donors? The predictive value of our best genes was about 80% based on the LDA model separatrix (Table 2). However, predictive models and separatrices can be fine tuned for clinical decision-making to either optimize sensitivity or specificity. An increase in sensitivity usually comes at the expense of a decrease in specificity, and vice versa. Given that the avoidance of GVHD is usually paramount, one would expect that a bias toward the best achievable sensitivity, allowing for the most reliable (or total) elimination of GVHD+ donors (while not eliminating too many donor candidates), would be clinically desirable (Figure 3 and Figure 4). Interestingly, PIA based on a pairwise gene-interaction model suggested that some genes have synergistic or competitive interactions that lead to increased predictive-model performance (Figure 4). This result also suggests that higher-order combinatorial searches beyond two genes could improve significantly the predictive performance of gene-expression profiling [30]. Thus, predictive models limited to a set of ten to 20 genes may achieve even greater than 80% accuracy and the robustness required for dependable AHCT donor selection. However, higher-order predictive variable combinations do require the support of many more samples to prevent overfitting of the model. Cogent assessment of this question will therefore necessitate expression profiling of genes identified herein in larger cohorts of participants. Thus, before gene-expression profiling can be widely used to guide clinical decision-making, it must be validated at other centers, in a wider range of patients. Similar to a recently reported index for post-AHCT assessment of GVHD severity [86], we envision predictive models based on pre-AHCT donor-expression profiling as an “evolving” evidence-based process for determining the risk of GVHD, to be recalibrated over time to account for changes in practice. As a corollary, a gene set that can identify strong alloresponders should also have predictive value for rejection of solid organ grafts. In summary, the results presented here could represent the basis of a breakthrough in transplantation medicine by helping selection of low-risk donors for AHCT, and tailoring the immunosuppressive regimens given to the recipient according to the risk of GVHD (AHCT) or rejection (solid organ).

## Supporting Information

Table S1 Genes Overexpressed in GVHD+ Relative to GVHD− Donors (22 KB XLS)

Table S2 Genes Repressed in GVHD+ Relative to GVHD− Donors (23 KB XLS)

Table S3 The Two Gene Sets That Were Used to Evaluate the Correlation between the Donor and Recipient Gene Expression Profiles They include the top 400 genes showing differential expression in GVHD+ versus GVHD− donors on day 0, combined with the top 400 genes showing differential expression in GVHD+ vs. GVHD− recipients on day 365.
(126 KB XLS)

Alternative Language Abstract S1 Translation of the Abstract into French by Claude Perreault (26 KB DOC)

### Accession Numbers

Microarray data in this paper are compliant to the minimum information about a microarray experiment (MIAME) criteria and are deposited at Gene Expression Omnibus (http://www.ncbi.nih.gov/geo; accession number GSE4624). The National Center for Biotechnology Information (http://www.ncbi.nih.gov) accession numbers for *TGIF* and *TGFBI* transcripts are NM_170695 and NM_000358, respectively. Those for all other transcripts used in this study are listed in Table S1 and Table S2.

## Abbreviations

aGVHD: acute graft-versus-host disease
AHCT: allogeneic hematopoietic cell transplantation
cGVHD: chronic graft-versus-host disease
CPIA: competitive predictive interaction analysis
GVHD: graft-versus-host disease
LDA: linear discriminant analysis
MHC: major histocompatibility complex
PIA: predictive interaction analysis
qRT-PCR: quantitative real-time PCR
SPIA: synergistic predictive interaction analysis
TGF-β: transforming growth factor-β
