# Supplementary material for: Prediction of Graft-Versus-Host Disease in Humans by Donor Gene-Expression Profiling
Source: PLoS Med. 2007 Jan 30;4(1):e23. doi: 10.1371/journal.pmed.0040023 (PMC1796639; doi:10.1371/journal.pmed.0040023)
Supplement: Table S3 — They include the top 400 genes showing differential expression in GVHD+ versus GVHD− donors on day 0, combined with the top 400 genes showing differential expression in GVHD+ vs. GVHD− recipients on day 365. (126 KB XLS) [file pmed.0040023.st003.pdf]

| Table S3. Top 400 genes differentially expressed in GVHD+ vs. GVHD- donors on day 0 and in GVHD+ vs. GVHD- recipients on day 365 | |
|---|---|
| day 0 | day 365 |
| 00018-EPHB2-EphB2 | 00012-ZNF224-zinc finger protein 224 |
| 00055-API5-apoptosis inhibitor 5 | 00015-DAPK1-death-associated protein kinase 1 |
| 00078-RAN-RAN, member RAS oncogene family | 00018-EPHB2-EphB2 |
| 00126-RAB25-RAB25, member RAS oncogene family | 00059-PAOX-polyamine oxidase (exo-N4-amino) |
| 00144-GNG13-guanine nucleotide binding protein (G protein), gamma 13 | 00063-PDCD11-programmed cell death 11 |
| 00148-MGC35285-hypothetical protein MGC35285 | 00100-NCOA3-nuclear receptor coactivator 3 |
| 00248-FMNL1-formin-like 1 | 00248-FMNL1-formin-like 1 |
| 00288-PSMA5-proteasome (prosome, macropain) subunit, alpha type, 5 | 00386-SERTAD1-SERTA domain containing 1 |
| 00346-BAG3-BCL2-associated athanogene 3 | 00390-GNAS-GNAS complex locus |
| 00504-SOCS5-suppressor of cytokine signaling 5 | 00393-NOS2A-nitric oxide synthase 2A (inducible, hepatocytes) |
| 00551-AKT2-v-akt murine thymoma viral oncogene homolog 2 | 00402-SCG2-secretogranin II (chromogranin C) |
| 00565-GAB1-GRB2-associated binding protein 1 | 00408-JRK-jerky homolog (mouse) |
| 00606-MT1G-metallothionein 1G | 00435-MAPT-microtubule-associated protein tau |
| 00666-TSBF1-tumor suppressor TSBF1 | 00436-DGCR14-DiGeorge syndrome critical region gene 14 |
| 00671-FLJ12985-hypothetical protein FLJ12985 | 00549-FY-Duffy blood group |
| 00692-SMAD3-MAD, mothers against decapentaplegic homolog 3 (Drosophila) | 00564-PLCB4-phospholipase C, beta 4 |
| 00734-XPO7-exportin 7 | 00566-SGK-serum/glucocorticoid regulated kinase |
| 00918-GULP1-GULP, engulfment adaptor PTB domain containing 1 | 00595-FRAP1-FK506 binding protein 12-rapamycin associated protein 1 |
| 00942-GRCA-likely ortholog of mouse gene rich cluster, A gene | 00692-MADH3-MAD, mothers against decapentaplegic homolog 3 (Drosophila) |
| 00956-FBLP-1-filamin-binding LIM protein-1 | 00941-COX4I1-cytochrome c oxidase subunit IV isoform 1 |
| 00983-CKS2-CDC28 protein kinase regulatory subunit 2 | 00974-DYRK4-dual-specificity tyrosine-(Y)-phosphorylation regulated kinase 4 |
| 01070-ANXA5-annexin A5 | 01042-GAB1-GRB2-associated binding protein 1 |
| 01100-TPM4-tropomyosin 4 | 01152-KDELR1-KDEL (Lys-Asp-Glu-Leu) endoplasmic reticulum protein retention receptor 1 |
| 01157-ATP6V1G1-ATPase, H+ transporting, lysosomal 13kDa, V1 subunit G isoform 1 | 01170-SAT-spermidine/spermine N1-acetyltransferase |
| 01193-RPS28-ribosomal protein S28 | 01193-RPS28-ribosomal protein S28 |
| 01210-SPARC-secreted protein, acidic, cysteine-rich (osteonectin) | 01213-TFEB-transcription factor EB |

| | |
|---|---|
| 01213-TFEB-transcription factor EB | 01266-GSN-gelsolin (amyloidosis, Finnish type) |
| 01278-SSB-Sjogren syndrome antigen B (autoantigen La) | 01336-SLC2A4RG-SLC2A4 regulator |
| 01306-DLX4-distal-less homeobox 4 | 01354-KIAA1285-KIAA1285 protein |
| 01307-ST18-suppression of tumorigenicity 18 (breast carcinoma) (zinc finger protein) | 01418-SULT1A1-sulfotransferase family, cytosolic, 1A, phenol-preferring, member 1 |
| 01386-PSMC4-proteasome (prosome, macropain) 26S subunit, ATPase, 4 | 01438-MRPL22-mitochondrial ribosomal protein L22 |
| 01431-FES-feline sarcoma oncogene | 01463-ELAC1-elaC homolog 1 (E. coli) |
| 01434-SIPA1-signal-induced proliferation-associated gene 1 | 01521-ASMTL-acetylserotonin O-methyltransferase-like |
| 01483-CCT6A-chaperonin containing TCP1, subunit 6A (zeta 1) | 01527-UBE2A-ubiquitin-conjugating enzyme E2A (RAD6 homolog) |
| 01499-CCNB2-cyclin B2 | 01528-BTBD14B-BTB (POZ) domain containing 14B |
| 01515-RPE-ribulose-5-phosphate-3-epimerase | 01715-RECK-reversion-inducing-cysteine-rich protein with kazal motifs |
| 01715-RECK-reversion-inducing-cysteine-rich protein with kazal motifs | 01757-ICAM3-intercellular adhesion molecule 3 |
| 01721-IFITM1-interferon induced transmembrane protein 1 (9-27) | 01819-PTPRE-protein tyrosine phosphatase, receptor type, E |
| 01723-CD79A-CD79A antigen (immunoglobulin-associated alpha) | 01839-MAD-MAX dimerization protein 1 |
| 01817-PHEMX-pan-hematopoietic expression | 01857-ASB8-ankyrin repeat and SOCS box-containing 8 |
| 01839-MAD-MAX dimerization protein 1 | 01888-ANKRD17-ankyrin repeat domain 17 |
| 01913-RHBDL2-rhomboid, veinlet-like 2 (Drosophila) | 02017-TTN-titin |
| 02007-GTPBP5-GTP binding protein 5 (putative) | 02027-MAST2-microtubule associated serine/threonine kinase 2 |
| 02043-NKIRAS2-NFKB inhibitor interacting Ras-like protein 2 | 02031-DP1-polyposis locus protein 1 |
| 02091-PDCD8-programmed cell death 8 (apoptosis-inducing factor) | 02080-PPHLN1-periphilin 1 |
| 02094-PSG9-pregnancy specific beta-1-glycoprotein 9 | 02174-HLA-DRB3-major histocompatibility complex, class II, DR beta 3 |
| 02186-GRWD1-glutamate-rich WD repeat containing 1 | 02175-PCM1-pericentriolar material 1 |
| 02193-KRT8-keratin 8 | 02225-ASH1L-ash1 (absent, small, or homeotic)-like (Drosophila) |
| 02240-CSNK2B-casein kinase 2, beta polypeptide | 02277-DELGEF-deafness locus associated putative guanine nucleotide exchange factor |
| 02358-ITGB4-integrin, beta 4 | 02358-ITGB4-integrin, beta 4 |
| 02368-MYCL1-v-myc myelocytomatosis viral oncogene homolog 1, lung carcinoma derived (avian) | 02393-NRIP1-nuclear receptor interacting protein 1 |
| 02410-RGN-regucalcin (senescence marker protein-30) | 02485-GEMIN4-gem (nuclear organelle) associated protein 4 |
| 02412-TLN2-talin 2 | 02522-TRIM41-tripartite motif-containing 41 |
| 02484-PILRB-paired immunoglobin-like type 2 receptor beta | 02554-RANGAP1-Ran GTPase activating protein 1 |
| 02502-ANK3-ankyrin 3, node of Ranvier (ankyrin G) | 02601-SULF2-sulfatase 2 |

| | |
|---|---|
| 02509-HDGF-hepatoma-derived growth factor (high-mobility group protein 1-like) | 02612-TP53I11-tumor protein p53 inducible protein 11 |
| 02567-PIGT-phosphatidylinositol glycan, class T | 02615-PFC-properdin P factor, complement |
| 02580-RHO-rhodopsin (opsin 2, rod pigment) (retinitis pigmentosa 4, autosomal dominant) | 02665-PRSS16-protease, serine, 16 (thymus) |
| 02628-SENP7-SUMO1/sentrin specific protease 7 | 02750-HOXA11-homeo box A11 |
| 02662-HBB-hemoglobin, beta | 02752-KDELR1-KDEL (Lys-Asp-Glu-Leu) endoplasmic reticulum protein retention receptor 1 |
| 02666-SPUVE-protease, serine, 23 | 02760-KAI1-kangai 1 (suppression of tumorigenicity 6, prostate; CD82 antigen (R2 leukocyte antigen, antigen detected by monoclonal and antibody IA4)) |
| 02720-DHCR24-24-dehydrocholesterol reductase | 02786-ATP5O-ATP synthase, H+ transporting, mitochondrial F1 complex, O subunit (oligomycin sensitivity conferring protein) |
| 02752-KDELR1-KDEL (Lys-Asp-Glu-Leu) endoplasmic reticulum protein retention receptor 1 | 02935-MBD3-methyl-CpG binding domain protein 3 |
| 02760-KAI1-kangai 1 (suppression of tumorigenicity 6, prostate; CD82 antigen (R2 leukocyte antigen, antigen detected by monoclonal and antibody IA4)) | 03239-FLJ21128-hypothetical protein FLJ21128 |
| 02786-ATP5O-ATP synthase, H+ transporting, mitochondrial F1 complex, O subunit (oligomycin sensitivity conferring protein) | 03260-SCYL1-SCY1-like 1 (S. cerevisiae) |
| 02844-COG7-component of oligomeric golgi complex 7 | 03262-SEC24A-SEC24 related gene family, member A (S. cerevisiae) |
| 02901-M6PRBP1-mannose-6-phosphate receptor binding protein 1 | 03279-MKRN2-makorin, ring finger protein, 2 |
| 02935-MBD3-methyl-CpG binding domain protein 3 | 03394-PIP5K1B-phosphatidylinositol-4-phosphate 5-kinase, type I, beta |
| 02993-ELP3-elongation protein 3 homolog (S. cerevisiae) | 03427-SIGIRR-single Ig IL-1R-related molecule |
| 03007-MT2A-metallothionein 2A | 03431-PHCA-phytoceramidase, alkaline |
| 03031-GSR-glutathione reductase | 03437-COLQ-collagen-like tail subunit (single strand of homotrimer) of asymmetric acetylcholinesterase |
| 03077-MTPN-myotrophin | 03443-TRIM32-tripartite motif-containing 32 |
| 03256-ZNF291-zinc finger protein 291 | 03484-MARS-methionine-tRNA synthetase |
| 03262-SEC24A-SEC24 related gene family, member A (S. cerevisiae) | 03485-BAP1-BRCA1 associated protein-1 (ubiquitin carboxy-terminal hydrolase) |
| 03330-PIASY-protein inhibitor of activated STAT protein PIASy | 03490-TGFA-transforming growth factor, alpha |
| 03343-MGAT3-mannosyl (beta-1,4-)-glycoprotein beta-1,4-N-acetylglucosaminyltransferase | 03545-SLC30A5-solute carrier family 30 (zinc transporter), member 5 |
| 03430-TAF13-TAF13 RNA polymerase II, TATA box binding protein (TBP)-associated factor, 18kDa | 03560-RAI3-retinoic acid induced 3 |
| 03443-TRIM32-tripartite motif-containing 32 | 03586-EPB41L4B-erythrocyte membrane protein band 4.1 like 4B |
| 03467-ANXA4-annexin A4 | 03590-ZNF75A-zinc finger protein 75a |

| | |
|---|---|
| 03481-GFAP-glial fibrillary acidic protein | 03678-PNUTL1-peanut-like 1 (Drosophila) |
| 03484-MARS-methionine-tRNA synthetase | 03683-RPL31-ribosomal protein L31 |
| 03560-RAI3-retinoic acid induced 3 | 03770-ILF3-interleukin enhancer binding factor 3, 90kDa |
| 03604-MYOHD1-myosin head domain containing 1 | 03793-MGC5178-hypothetical protein MGC5178 |
| 03676-CALM2-calmodulin 2 (phosphorylase kinase, delta) | 03824-ACTR1A-ARP1 actin-related protein 1 homolog A, centractin alpha (yeast) |
| 03705-ZNF-kaiso-kaiso | 03825-RAB13-RAB13, member RAS oncogene family |
| 03759-COX5A-cytochrome c oxidase subunit Va | 03865-CLOCK-clock homolog (mouse) |
| 03770-ILF3-interleukin enhancer binding factor 3, 90kDa | 03898-PRDX4-peroxiredoxin 4 |
| 03808-ACADSB-acyl-Coenzyme A dehydrogenase, short/branched chain | 03927-ATF6-activating transcription factor 6 |
| 03825-RAB13-RAB13, member RAS oncogene family | 03954-CLTB-clathrin, light polypeptide (Lcb) |
| 03896-DNASE2-deoxyribonuclease II, lysosomal | 04006-CKAP4-cytoskeleton-associated protein 4 |
| 03898-PRDX4-peroxiredoxin 4 | 04045-STAU-staufen, RNA binding protein (Drosophila) |
| 03917-TNFRSF7-tumor necrosis factor receptor superfamily, member 7 | 04119-AF5Q31-ALL1 fused gene from 5q31 |
| 03927-ATF6-activating transcription factor 6 | 04145-SLC6A1-solute carrier family 6 (neurotransmitter transporter, GABA), member 1 |
| 03928-PPIE-peptidylprolyl isomerase E (cyclophilin E) | 04209-RAB26-RAB26, member RAS oncogene family |
| 03945-TNFRSF1B-tumor necrosis factor receptor superfamily, member 1B | 04268-H6PD-hexose-6-phosphate dehydrogenase (glucose 1-dehydrogenase) |
| 04012-LAMP2-lysosomal-associated membrane protein 2 | 04556-VPS28-vacuolar protein sorting 28 (yeast) |
| 04072-COL6A1-collagen, type VI, alpha 1 | 04606-STRN4-striatin, calmodulin binding protein 4 |
| 04131-ANXA5-annexin A5 | 04616-ECM1-extracellular matrix protein 1 |
| 04145-SLC6A1-solute carrier family 6 (neurotransmitter transporter, GABA), member 1 | 04668-FBXL10-F-box and leucine-rich repeat protein 10 |
| 04151-CD24-CD24 antigen (small cell lung carcinoma cluster 4 antigen) | 04721-ASMTL-acetylserotonin O-methyltransferase-like |
| 04209-RAB26-RAB26, member RAS oncogene family | 04742-NDUFS1-NADH dehydrogenase (ubiquinone) Fe-S protein 1, 75kDa (NADH-coenzyme Q reductase) |
| 04296-GPSN2-glycoprotein, synaptic 2 | 04745-FLJ13352-hypothetical protein FLJ13352 |
| 04354-THRAP6-thyroid hormone receptor associated protein 6 | 05016-EIF3S4-eukaryotic translation initiation factor 3, subunit 4 delta, 44kDa |
| 04370-MPG-N-methylpurine-DNA glycosylase | 05071-GNA14-guanine nucleotide binding protein (G protein), alpha 14 |
| 04418-GRP58-glucose regulated protein, 58kDa | 05093-RPS3A-ribosomal protein S3A |
| 04434-CENTA1-centaurin, alpha 1 | 05200-SILV-silver homolog (mouse) |
| 04550-ATBF1-AT-binding transcription factor 1 | 05373-ZFP36L1-zinc finger protein 36, C3H type-like 1 |

| | |
|---|---|
| 04601-APEX2-APEX nuclease (apurinic/apyrimidinic endonuclease) 2 | 05463-CDW52-CDW52 antigen (CAMPATH-1 antigen) |
| 04721-ASMTL-acetylserotonin O-methyltransferase-like | 05553-CDC34-cell division cycle 34 |
| 04842-NDRG3-NDRG family member 3 | 05601-CABIN1-calcineurin binding protein 1 |
| 04924-RNH-ribonuclease/angiogenin inhibitor | 05623-SPOCK2-sparc/osteonectin, cwcv and kazal-like domains proteoglycan (testican) 2 |
| 04941-TRPV6-transient receptor potential cation channel, subfamily V, member 6 | 05629-ILF3-interleukin enhancer binding factor 3, 90kDa |
| 04993-ROCK1-Rho-associated, coiled-coil containing protein kinase 1 | 05666-RAD23B-RAD23 homolog B (S. cerevisiae) |
| 05008-GMNN-geminin, DNA replication inhibitor | 05674-CKLF-chemokine-like factor |
| 05138-PRKAR1B-protein kinase, cAMP-dependent, regulatory, type I, beta | 05680-HIRA-HIR histone cell cycle regulation defective homolog A (S. cerevisiae) |
| 05195-EBI2-Epstein-Barr virus induced gene 2 (lymphocyte-specific G protein-coupled receptor) | 05731-C21orf63-chromosome 21 open reading frame 63 |
| 05232-CD63-CD63 antigen (melanoma 1 antigen) | 05761-GALT-galactose-1-phosphate uridylyltransferase |
| 05314-SNAP23-synaptosomal-associated protein, 23kDa | 05775-GLUL-glutamate-ammonia ligase (glutamine synthase) |
| 05407-MPHOSPH1-M-phase phosphoprotein 1 | 05811-FTH1-ferritin, heavy polypeptide 1 |
| 05484-CSRP2-cysteine and glycine-rich protein 2 | 05823-FER1L3-fer-1-like 3, myoferlin (C. elegans) |
| 05506-AHSG-alpha-2-HS-glycoprotein | 05827-SRP14-signal recognition particle 14kDa (homologous Alu RNA binding protein) |
| 05554-HSD11B1-hydroxysteroid (11-beta) dehydrogenase 1 | 05889-ERP70-protein disulfide isomerase related protein (calcium-binding protein, intestinal-related) |
| 05587-EPLIN-epithelial protein lost in neoplasm beta | 05897-IL11RA-interleukin 11 receptor, alpha |
| 05652-TM6SF1-transmembrane 6 superfamily member 1 | 05936-C10orf9-chromosome 10 open reading frame 9 |
| 05664-TOMM20-translocase of outer mitochondrial membrane 20 homolog (yeast) | 05943-SHARP-SMART/HDAC1 associated repressor protein |
| 05666-RAD23B-RAD23 homolog B (S. cerevisiae) | 05969-DDX5-DEAD (Asp-Glu-Ala-Asp) box polypeptide 5 |
| 05674-CKLF-chemokine-like factor | 05999-NEDD8-neural precursor cell expressed, developmentally down-regulated 8 |
| 05721-GPX3-glutathione peroxidase 3 (plasma) | 06080-ATP13A-ATPase type 13A |
| 05763-BST1-bone marrow stromal cell antigen 1 | 06092-ZNF384-zinc finger protein 384 |
| 05823-FER1L3-fer-1-like 3, myoferlin (C. elegans) | 06186-RCN3-reticulocalbin 3, EF-hand calcium binding domain |
| 06001-GTF3C4-general transcription factor IIIC, polypeptide 4, 90kDa | 06196-HLA-B-major histocompatibility complex, class I, B |
| 06060-TMEM8-transmembrane protein 8 (five membrane-spanning domains) | 06217-PTGS1-prostaglandin-endoperoxide synthase 1 (prostaglandin G/H synthase and cyclooxygenase) |
| 06080-ATP13A-ATPase type 13A | 06257-SUI1-putative translation initiation factor |
| 06148-RAB9P40-Rab9 effector p40 | 06277-DustyPK-dusty protein kinase |
| 06149-CD81-CD81 antigen (target of antiproliferative antibody 1) | 06286-ZFP36L2-zinc finger protein 36, C3H type-like 2 |

| | |
|---|---|
| 06177-SNRPN-small nuclear ribonucleoprotein polypeptide N | 06320-SF3B2-splicing factor 3b, subunit 2, 145kDa |
| 06315-KPTN-kaptin (actin binding protein) | 06345-LMOD1-leiomodin 1 (smooth muscle) |
| 06352-PDE7B-phosphodiesterase 7B | 06466-GTL3-likely ortholog of mouse gene trap locus 3 |
| 06380-GAPD-glyceraldehyde-3-phosphate dehydrogenase | 06497-PGPEP1-pyroglutamyl-peptidase I |
| 06412-USP28-ubiquitin specific protease 28 | 06521-COL5A3-collagen, type V, alpha 3 |
| 06465-APOC1-apolipoprotein C-I | 06554-TRAPPC1-trafficking protein particle complex 1 |
| 06497-PGPEP1-pyroglutamyl-peptidase I | 06608-GMNN-geminin, DNA replication inhibitor |
| 06549-CEP2-centrosomal protein 2 | 06636-NR4A3-nuclear receptor subfamily 4, group A, member 3 |
| 06560-PEPD-peptidase D | 06672-RPS3A-ribosomal protein S3A |
| 06565-SAE1-SUMO-1 activating enzyme subunit 1 | 06726-ZNF219-zinc finger protein 219 |
| 06568-TMEM1-transmembrane protein 1 | 06842-TOB2-transducer of ERBB2, 2 |
| 06593-ROCK1-Rho-associated, coiled-coil containing protein kinase 1 | 06891-LOC57019-hypothetical protein LOC57019 |
| 06608-GMNN-geminin, DNA replication inhibitor | 06925-KDELR1-KDEL (Lys-Asp-Glu-Leu) endoplasmic reticulum protein retention receptor 1 |
| 06664-MGC13138-hypothetical protein MGC13138 | 07001-DSC2-desmocollin 2 |
| 06818-XPR1-xenotropic and polytropic retrovirus receptor | 07008-URKL1-uridine kinase-like 1 |
| 06879-NEDL1-HECT type E3 ubiquitin ligase | 07018-RPS6-ribosomal protein S6 |
| 07003-PRKACA-protein kinase, cAMP-dependent, catalytic, alpha | 07119-RPS27L-ribosomal protein S27-like |
| 07028-PRKAA1-protein kinase, AMP-activated, alpha 1 catalytic subunit | 07129-HLA-DRB3-major histocompatibility complex, class II, DR beta 3 |
| 07129-HLA-DRB3-major histocompatibility complex, class II, DR beta 3 | 07142-HK3-hexokinase 3 (white cell) |
| 07151-IL1R1-interleukin 1 receptor, type I | 07211-NCOR1-nuclear receptor co-repressor 1 |
| 07211-NCOR1-nuclear receptor co-repressor 1 | 07264-SLC9A5-solute carrier family 9 (sodium/hydrogen exchanger), isoform 5 |
| 07346-RAB6A-RAB6A, member RAS oncogene family | 07274-CKLF-chemokine-like factor |
| 07357-TCF7L1-transcription factor 7-like 1 (T-cell specific, HMG-box) | 07322-TLE2-transducin-like enhancer of split 2 (E(sp1) homolog, Drosophila) |
| 07359-NUMA1-nuclear mitotic apparatus protein 1 | 07344-FLJ23469-hypothetical protein FLJ23469 |
| 07366-CAMKK1-calcium/calmodulin-dependent protein kinase kinase 1, alpha | 07346-RAB6A-RAB6A, member RAS oncogene family |
| 07408-AGMAT-agmatine ureohydrolase (agmatinase) | 07366-CAMKK1-calcium/calmodulin-dependent protein kinase kinase 1, alpha |
| 07469-VIM-vimentin | 07469-VIM-vimentin |
| 07508-NR2F2-nuclear receptor subfamily 2, group F, member 2 | 07496-FMN2-formin 2 |
| 07520-ACTR1B-ARP1 actin-related protein 1 homolog B, centractin beta (yeast) | 07520-ACTR1B-ARP1 actin-related protein 1 homolog B, centractin beta (yeast) |
| 07568-SC4MOL-sterol-C4-methyl oxidase-like | 07526-TLE2-transducin-like enhancer of split 2 (E(sp1) homolog, Drosophila) |

| | |
|---|---|
| 07569-SAS-sarcoma amplified sequence | 07602-YWHAQ-tyrosine 3-monooxygenase/tryptophan 5-monooxygenase activation protein, theta polypeptide |
| 07598-DHCR24-24-dehydrocholesterol reductase | 07657-PLOD-procollagen-lysine, 2-oxoglutarate 5-dioxygenase (lysine hydroxylase, Ehlers-Danlos syndrome type VI) |
| 07599-RAB2-RAB2, member RAS oncogene family | 07701-GNS-glucosamine (N-acetyl)-6-sulfatase (Sanfilippo disease IIID) |
| 07602-YWHAQ-tyrosine 3-monooxygenase/tryptophan 5-monooxygenase activation protein, theta polypeptide | 07750-NBL1-neuroblastoma, suppression of tumorigenicity 1 |
| 07704-HOXB5-homeo box B5 | 07759-IMMT-inner membrane protein, mitochondrial (mitofilin) |
| 07776-NFKB2-nuclear factor of kappa light polypeptide gene enhancer in B-cells 2 (p49/p100) | 07781-CD68-CD68 antigen |
| 07781-CD68-CD68 antigen | 07788-DPF2-D4, zinc and double PHD fingers family 2 |
| 07812-DAP-death-associated protein | 07789-PPP2R5C-protein phosphatase 2, regulatory subunit B (B56), gamma isoform |
| 07828-PTGER4-prostaglandin E receptor 4 (subtype EP4) | 07809-SLC9A1-solute carrier family 9 (sodium/hydrogen exchanger), isoform 1 (antiporter, Na+/H+, amiloride sensitive) |
| 07835-PSMB4-proteasome (prosome, macropain) subunit, beta type, 4 | 07827-ANXA11-annexin A11 |
| 07926-MINK-misshapen/NIK-related kinase | 07898-IFI30-interferon, gamma-inducible protein 30 |
| 07950-SSRP1-structure specific recognition protein 1 | 07913-C5-complement component 5 |
| 08141-EPB49-erythrocyte membrane protein band 4.9 (dematin) | 07950-SSRP1-structure specific recognition protein 1 |
| 08197-CYP39A1-cytochrome P450, family 39, subfamily A, polypeptide 1 | 08041-NRG1-neuregulin 1 |
| 08257-QRSL1-glutaminyl-tRNA synthase (glutamine-hydrolyzing)-like 1 | 08125-FN1-fibronectin 1 |
| 08346-POLR2J-polymerase (RNA) II (DNA directed) polypeptide J, 13.3kDa | 08141-EPB49-erythrocyte membrane protein band 4.9 (dematin) |
| 08417-TAGLN2-transgelin 2 | 08150-JAK1-Janus kinase 1 (a protein tyrosine kinase) |
| 08434-MORF4L1-mortality factor 4 like 1 | 08163-IL16-interleukin 16 (lymphocyte chemoattractant factor) |
| 08512-TREX1-three prime repair exonuclease 1 | 08230-PPP2CB-protein phosphatase 2 (formerly 2A), catalytic subunit, beta isoform |
| 08568-LOC124245-hypothetical protein BC001584 | 08265-BENE-BENE protein |
| 08778-TBCD-tubulin-specific chaperone d | 08272-RPS3A-ribosomal protein S3A |
| 08877-RNPEPL1-arginyl aminopeptidase (aminopeptidase B)-like 1 | 08329-TBXA2R-thromboxane A2 receptor |
| 08942-DNM1-dynamin 1 | 08343-UBE2V2-ubiquitin-conjugating enzyme E2 variant 2 |
| 08974-UAP1-UDP-N-acteylglucosamine pyrophosphorylase 1 | 08417-TAGLN2-transgelin 2 |
| 09016-TGFBRAP1-transforming growth factor, beta receptor associated protein 1 | 08433-NTRK3-neurotrophic tyrosine kinase, receptor, type 3 |

| | |
|---|---|
| 09069-VIM-vimentin | 08543-FLJ22021-hypothetical protein FLJ22021 |
| 09112-SEL1L-sel-1 suppressor of lin-12-like (C. elegans) | 08654-DAZAP1-DAZ associated protein 1 |
| 09117-ADH6-alcohol dehydrogenase 6 (class V) | 08657-RQCD1-RCD1 required for cell differentiation1 homolog (S. pombe) |
| 09132-RPL31-ribosomal protein L31 | 08692-TA-PP2C-T-cell activation protein phosphatase 2C |
| 09137-EIF4G2-eukaryotic translation initiation factor 4 gamma, 2 | 08696-ENG-endoglin (Osler-Rendu-Weber syndrome 1) |
| 09159-VRK3-vaccinia related kinase 3 | 08747-UBE2V2-ubiquitin-conjugating enzyme E2 variant 2 |
| 09177-C6orf69-chromosome 6 open reading frame 69 | 08786-IL8-interleukin 8 |
| 09206-MOBP-myelin-associated oligodendrocyte basic protein | 08850-PFN2-profilin 2 |
| 09232-CD63-CD63 antigen (melanoma 1 antigen) | 08938-GTF3A-general transcription factor IIIA |
| 09278-PGPL-pseudoautosomal GTP-binding protein-like | 08942-DNM1-dynamin 1 |
| 09291-ZNF205-zinc finger protein 205 | 08974-UAP1-UDP-N-acteylglucosamine pyrophosphorylase 1 |
| 09304-RAB40C-RAB40C, member RAS oncogene family | 09002-GSTA3-glutathione S-transferase A3 |
| 09397-PDE2A-phosphodiesterase 2A, cGMP-stimulated | 09069-VIM-vimentin |
| 09488-TGOLN2-trans-golgi network protein 2 | 09086-RPL41-ribosomal protein L41 |
| 09497-FOXJ1-forkhead box J1 | 09132-RPL31-ribosomal protein L31 |
| 09503-KNS2-kinesin 2 60/70kDa | 09206-MOBP-myelin-associated oligodendrocyte basic protein |
| 09571-TPM1-tropomyosin 1 (alpha) | 09298-USP7-ubiquitin specific protease 7 (herpes virus-associated) |
| 09678-TMEM9-transmembrane protein 9 | 09397-PDE2A-phosphodiesterase 2A, cGMP-stimulated |
| 09878-VEGF-vascular endothelial growth factor | 09429-LASP1-LIM and SH3 protein 1 |
| 09911-CYP4F12-cytochrome P450, family 4, subfamily F, polypeptide 12 | 09599-EGFL3-EGF-like-domain, multiple 3 |
| 10053-JPH3-junctophilin 3 | 09641-C16orf40-chromosome 16 open reading frame 40 |
| 10071-FKSG44-hypothetical protein FKSG44 | 09760-NDUFS7-NADH dehydrogenase (ubiquinone) Fe-S protein 7, 20kDa (NADH-coenzyme Q reductase) |
| 10114-TCEB3BP1-transcription elongation factor B polypeptide 3 binding protein 1 | 09872-SEMA3F-sema domain, immunoglobulin domain (Ig), short basic domain, secreted, (semaphorin) 3F |
| 10164-LOC132241-hypothetical protein LOC132241 | 09887-DPT-dermatopontin |
| 10213-RAMP3-receptor (calcitonin) activity modifying protein 3 | 09996-TADA3L-transcriptional adaptor 3 (NGG1 homolog, yeast)-like |
| 10260-BCAP31-B-cell receptor-associated protein 31 | 09997-MDS028-uncharacterized hematopoietic stem/progenitor cells protein MDS028 |
| 10266-APOB-apolipoprotein B (including Ag(x) antigen) | 10011-DDOST-dolichyl-diphosphooligosaccharide-protein glycosyltransferase |

| | |
|---|---|
| 10272-RRAGD-Ras-related GTP binding D | 10104-ZNF444-zinc finger protein 444 |
| 10295-IGFBP6-insulin-like growth factor binding protein 6 | 10170-MGC16943-similar to RIKEN cDNA 4933424N09 gene |
| 10461-SNRPD3-small nuclear ribonucleoprotein D3 polypeptide 18kDa | 10172-RPL23A-ribosomal protein L23a |
| 10542-SYT5-synaptotagmin V | 10177-FY-Duffy blood group |
| 10564-SCML1-sex comb on midleg-like 1 (Drosophila) | 10261-BBS1-Bardet-Biedl syndrome 1 |
| 10597-MCM3AP-MCM3 minichromosome maintenance deficient 3 (S. cerevisiae) associated protein | 10295-IGFBP6-insulin-like growth factor binding protein 6 |
| 10600-SOX12-SRY (sex determining region Y)-box 12 | 10344-ISG20-interferon stimulated gene 20kDa |
| 10630-PPARD-peroxisome proliferative activated receptor, delta | 10454-PPP2R4-protein phosphatase 2A, regulatory subunit B' (PR 53) |
| 10650-MBNL2-muscleblind-like 2 (Drosophila) | 10457-PRKCG-protein kinase C, gamma |
| 10832-CAPN10-calpain 10 | 10542-SYT5-synaptotagmin V |
| 10874-CARD14-caspase recruitment domain family, member 14 | 10597-MCM3AP-MCM3 minichromosome maintenance deficient 3 (S. cerevisiae) associated protein |
| 10877-CBFA2T2-core-binding factor, runt domain, alpha subunit 2; translocated to, 2 | 10688-LOC134147-hypothetical protein BC001573 |
| 10987-PRG2-proteoglycan 2, bone marrow (natural killer cell activator, eosinophil granule major basic protein) | 10755-COL18A1-collagen, type XVIII, alpha 1 |
| 11001-C20orf121-chromosome 20 open reading frame 121 | 10832-CAPN10-calpain 10 |
| 11023-RFXANK-regulatory factor X-associated ankyrin-containing protein | 10850-DRPLA-dentatorubral-pallidoluysian atrophy (atrophin-1) |
| 11029-DKFZP566E144-small fragment nuclease | 10853-NBEA-neurobeachin |
| 11091-HEXA-hexosaminidase A (alpha polypeptide) | 10874-CARD14-caspase recruitment domain family, member 14 |
| 11107-UBE2H-ubiquitin-conjugating enzyme E2H (UBC8 homolog, yeast) | 10888-BRD2-bromodomain containing 2 |
| 11126-DDX54-DEAD (Asp-Glu-Ala-Asp) box polypeptide 54 | 10994-RBM10-RNA binding motif protein 10 |
| 11127-GGTLA1-gamma-glutamyltransferase-like activity 1 | 11000-DGAT1-diacylglycerol O-acyltransferase homolog 1 (mouse) |
| 11154-TEAD1-TEA domain family member 1 (SV40 transcriptional enhancer factor) | 11001-C20orf121-chromosome 20 open reading frame 121 |
| 11162-HS3ST4-heparan sulfate (glucosamine) 3-O-sulfotransferase 4 | 11029-DKFZP566E144-small fragment nuclease |
| 11200-HCRTR1-hypocretin (orexin) receptor 1 | 11122-PTTG1IP-pituitary tumor-transforming 1 interacting protein |
| 11243-TIPARP-TCDD-inducible poly(ADP-ribose) polymerase | 11142-DSIPI-delta sleep inducing peptide, immunoreactor |
| 11263-MASP1-mannan-binding lectin serine protease 1 (C4/C2 activating component of Ra-reactive factor) | 11202-RGS19-regulator of G-protein signalling 19 |
| 11332-FLJ11856-putative G-protein coupled receptor GPCR41 | 11275-C17-cytokine-like protein C17 |
| 11333-RGS12-regulator of G-protein signalling | 11276-FOSL1-FOS-like antigen 1 |

| | |
|---|---|
| 12 | |
| 11352-NEUROD6-neurogenic differentiation 6 | 11332-FLJ11856-putative G-protein coupled receptor GPCR41 |
| 11381-RHOT1-ras homolog gene family, member T1 | 11348-IFITM1-interferon induced transmembrane protein 1 (9-27) |
| 11409-MYL4-myosin, light polypeptide 4, alkali; atrial, embryonic | 11381-RHOT1-ras homolog gene family, member T1 |
| 11467-SPUVE-protease, serine, 23 | 11451-RPS3A-ribosomal protein S3A |
| 11484-HSPB1-heat shock 27kDa protein 1 | 11552-C2orf3-chromosome 2 open reading frame 3 |
| 11539-CSTB-cystatin B (stefin B) | 11557-DHRS10-dehydrogenase/reductase (SDR family) member 10 |
| 11554-SLC2A10-solute carrier family 2 (facilitated glucose transporter), member 10 | 11573-Dnaja4-DnaJ (Hsp40) homolog, subfamily A, member 4 |
| 11742-CHERP-calcium homeostasis endoplasmic reticulum protein | 11578-FN1-fibronectin 1 |
| 11784-ZF-HCF-binding transcription factor Zhangfei | 11586-SMARCE1-SWI/SNF related, matrix associated, actin dependent regulator of chromatin, subfamily e, member 1 |
| 11787-F2-coagulation factor II (thrombin) | 11605-ALDH2-aldehyde dehydrogenase 2 family (mitochondrial) |
| 11796-HEL308-DNA helicase HEL308 | 11741-RPL41-ribosomal protein L41 |
| 11807-DUSP22-dual specificity phosphatase 22 | 11742-CHERP-calcium homeostasis endoplasmic reticulum protein |
| 11824-CSDA-cold shock domain protein A | 11777-TUBB4-tubulin, beta, 4 |
| 11826-SNX15-sorting nexin 15 | 11796-HEL308-DNA helicase HEL308 |
| 11902-CDH11-cadherin 11, type 2, OB-cadherin (osteoblast) | 11824-CSDA-cold shock domain protein A |
| 11928-IL1R1-interleukin 1 receptor, type I | 11826-SNX15-sorting nexin 15 |
| 12071-TM4SF8-transmembrane 4 superfamily member 8 | 11830-CD44-CD44 antigen (homing function and Indian blood group system) |
| 12121-SLC27A1-solute carrier family 27 (fatty acid transporter), member 1 | 11880-NR1D1-nuclear receptor subfamily 1, group D, member 1 |
| 12155-DNB5-deleted in neuroblastoma 5 | 11925-ZNF606-zinc finger protein 606 |
| 12188-TERE1-transitional epithelia response protein | 11929-EHD2-EH-domain containing 2 |
| 12210-RANBP2-RAN binding protein 2 | 12046-DDOST-dolichyl-diphosphooligosaccharide-protein glycosyltransferase |
| 12221-APOE-apolipoprotein E | 12071-TM4SF8-transmembrane 4 superfamily member 8 |
| 12282-RFC4-replication factor C (activator 1) 4, 37kDa | 12175-FLJ14360-hypothetical protein FLJ14360 |
| 12350-PAPSS1-3'-phosphoadenosine 5'-phosphosulfate synthase 1 | 12188-TERE1-transitional epithelia response protein |
| 12355-COL18A1-collagen, type XVIII, alpha 1 | 12199-BDH-3-hydroxybutyrate dehydrogenase (heart, mitochondrial) |
| 12357-CAV1-caveolin 1, caveolae protein, 22kDa | 12204-GSTO1-glutathione S-transferase omega 1 |
| 12378-ARGBP2-Arg/Abl-interacting protein ArgBP2 | 12214-NS-nucleostemin |
| 12425-IMPA2-inositol(myo)-1(or 4)-monophosphatase 2 | 12225-B3GALT4-UDP-Gal:betaGlcNAc beta 1,3-galactosyltransferase, polypeptide 4 |

| | |
|---|---|
| 12493-GABARAP-GABA(A) receptor-associated protein | 12286-CHAF1A-chromatin assembly factor 1, subunit A (p150) |
| 12569-ZAK-sterile alpha motif and leucine zipper containing kinase AZK | 12288-MLF1-myeloid leukemia factor 1 |
| 12599-SULT1E1-sulfotransferase family 1E, estrogen-preferring, member 1 | 12378-ARGBP2-Arg/Abl-interacting protein ArgBP2 |
| 12624-P2RX7-purinergic receptor P2X, ligand-gated ion channel, 7 | 12425-IMPA2-inositol(myo)-1(or 4)-monophosphatase 2 |
| 12627-SOX4-SRY (sex determining region Y)-box 4 | 12426-FNBP2-formin binding protein 2 |
| 12629-LASP1-LIM and SH3 protein 1 | 12503-DDX3X-DEAD (Asp-Glu-Ala-Asp) box polypeptide 3, X-linked |
| 12639-CSNK2B-casein kinase 2, beta polypeptide | 12599-SULT1E1-sulfotransferase family 1E, estrogen-preferring, member 1 |
| 12729-SCARB1-scavenger receptor class B, member 1 | 12620-HLA-DRB3-major histocompatibility complex, class II, DR beta 3 |
| 12799-TNXB-tenascin XB | 12625-MCM4-MCM4 minichromosome maintenance deficient 4 (S. cerevisiae) |
| 12859-Cbx5-chromobox homolog 5 (Drosophila HP1a) | 12626-RAB39B-RAB39B, member RAS oncogene family |
| 12881-MKI67IP-MKI67 (FHA domain) interacting nucleolar phosphoprotein | 12720-ATP6V1B2-ATPase, H+ transporting, lysosomal 56/58kDa, V1 subunit B, isoform 2 |
| 12988-ERCC1-excision repair cross-complementing rodent repair deficiency, complementation group 1 (includes overlapping antisense sequence) | 12723-GLI4-GLI-Kruppel family member GLI4 |
| 13035-MCC-mutated in colorectal cancers | 12785-DDX48-DEAD (Asp-Glu-Ala-Asp) box polypeptide 48 |
| 13350-NPC2-Niemann-Pick disease, type C2 | 12859-Cbx5-chromobox homolog 5 (Drosophila HP1a) |
| 13393-PARG-poly (ADP-ribose) glycohydrolase | 12881-MKI67IP-MKI67 (FHA domain) interacting nucleolar phosphoprotein |
| 13547-TRIM29-tripartite motif-containing 29 | 13010-MAT1A-methionine adenosyltransferase I, alpha |
| 13552-DAD1-defender against cell death 1 | 13018-CBFA2T3-core-binding factor, runt domain, alpha subunit 2; translocated to, 3 |
| 13617-YY1-YY1 transcription factor | 13024-SP100-nuclear antigen Sp100 |
| 13709-PCBP1-poly(rC) binding protein 1 | 13094-RPL31-ribosomal protein L31 |
| 13750-LOC221955-KCCR13L | 13204-PIP3-E-phosphoinositide-binding protein PIP3-E |
| 13757-RALB-v-ral simian leukemia viral oncogene homolog B (ras related; GTP binding protein) | 13316-CGA-glycoprotein hormones, alpha polypeptide |
| 13771-QSCN6L1-quiescin Q6-like 1 | 13356-HLA-E-major histocompatibility complex, class I, E |
| 13866-FN1-fibronectin 1 | 13373-KIAA0420-KIAA0420 gene product |
| 14005-MGC24039-hypothetical protein MGC24039 | 13380-CLSTN3-calsyntenin 3 |
| 14042-RBM8A-RNA binding motif protein 8A | 13393-PARG-poly (ADP-ribose) glycohydrolase |
| 14177-LONP-peroxisomal lon protease | 13419-GTPBP1-GTP binding protein 1 |
| 14178-DPP6-dipeptidylpeptidase 6 | 13423-ESD-esterase D/formylglutathione hydrolase |
| 14201-NSEP1-nuclease sensitiv e element | 13547-TRIM29-tripartite motif-containing 29 |

| | |
|---|---|
| binding protein 1 | |
| 14296-PLTP-phospholipid transfer protein | 13562-ACMSD-aminocarboxymuconate semialdehyde decarboxylase |
| 14300-PILRB-paired immunoglobin-like type 2 receptor beta | 13671-RHCE-Rhesus blood group, CcEe antigens |
| 14356-POU2F1-POU domain, class 2, transcription factor 1 | 13718-FXR2-fragile X mental retardation, autosomal homolog 2 |
| 14486-SLC4A5-solute carrier family 4, sodium bicarbonate cotransporter, member 5 | 13798-KIF12-kinesin family member 12 |
| 14528-DUSP1-dual specificity phosphatase 1 | 13837-DUSP1-dual specificity phosphatase 1 |
| 14549-EVI5-ecotropic viral integration site 5 | 13915-REV1L-REV1-like (yeast) |
| 14623-ATP5G3-ATP synthase, H+ transporting, mitochondrial F0 complex, subunit c (subunit 9) isoform 3 | 13949-PCSK7-proprotein convertase subtilisin/kexin type 7 |
| 14628-MAN2A2-mannosidase, alpha, class 2A, member 2 | 13988-WRN-Werner syndrome |
| 14665-PNUTL2-peanut-like 2 (Drosophila) | 14016-MYH10-myosin, heavy polypeptide 10, non-muscle |
| 14696-BSPRY-B-box and SPRY domain containing | 14018-KIF2-kinesin heavy chain member 2 |
| 14825-NME1-non-metastatic cells 1, protein (NM23A) expressed in | 14042-RBM8A-RNA binding motif protein 8A |
| 14941-RPS28-ribosomal protein S28 | 14068-IGHMBP2-immunoglobulin mu binding protein 2 |
| 14944-CELSR2-cadherin, EGF LAG seven-pass G-type receptor 2 (flamingo homolog, Drosophila) | 14129-TMF1-TATA element modulatory factor 1 |
| 15027-CCND1-cyclin D1 (PRAD1: parathyroid adenomatosis 1) | 14156-PER1-period homolog 1 (Drosophila) |
| 15028-CIAO1-WD40 protein Ciao1 | 14203-ZNF562-zinc finger protein 562 |
| 15079-ARPP-19-cyclic AMP phosphoprotein, 19 kD | 14208-LSS-lanosterol synthase (2,3-oxidosqualene-lanosterol cyclase) |
| 15148-SLC25A5-solute carrier family 25 (mitochondrial carrier; adenine nucleotide translocator), member 5 | 14224-PTK9-PTK9 protein tyrosine kinase 9 |
| 15216-UNC5A-unc-5 homolog A (C. elegans) | 14328-COPS8-COP9 constitutive photomorphogenic homolog subunit 8 (Arabidopsis) |
| 15229-NUP155-nucleoporin 155kDa | 14429-MGC45419-Similar to calcium/calmodulin-dependent protein kinase 1, beta |
| 15313-SKP2-S-phase kinase-associated protein 2 (p45) | 14462-WAS-Wiskott-Aldrich syndrome (eczema-thrombocytopenia) |
| 15322-CD79B-CD79B antigen (immunoglobulin-associated beta) | 14521-HLA-DQB1-major histocompatibility complex, class II, DQ beta 1 |
| 15341-ADORA1-adenosine A1 receptor | 14524-NCOA6IP-nuclear receptor coactivator 6 interacting protein |
| 15346-IDS-iduronate 2-sulfatase (Hunter syndrome) | 14529-TCL1A-T-cell leukemia/lymphoma 1A |
| 15363-CTSE-cathepsin E | 14536-ZYX-zyxin |
| 15416-ADD1-adducin 1 (alpha) | 14633-DIA1-diaphorase (NADH) (cytochrome b-5 reductase) |
| 15440-SQRDL-sulfide quinone reductase-like | 14688-EVPL-envoplakin |

| | |
|---|---|
| (yeast) | |
| 15461-DOK2-docking protein 2, 56kDa | 14798-RPL41-ribosomal protein L41 |
| 15562-CDC25B-cell division cycle 25B | 14826-CASC3-cancer susceptibility candidate 3 |
| 15656-CRTAC1-cartilage acidic protein 1 | 14897-BG1-lipidosin |
| 15678-CALM2-calmodulin 2 (phosphorylase kinase, delta) | 14906-PGR1-T-cell activation protein |
| 15719-ACOX1-acyl-Coenzyme A oxidase 1, palmitoyl | 14926-CHST5-carbohydrate (N-acetylglucosamine 6-O) sulfotransferase 5 |
| 15753-EEF1E1-eukaryotic translation elongation factor 1 epsilon 1 | 14941-RPS28-ribosomal protein S28 |
| 15799-UXS1-UDP-glucuronate decarboxylase 1 | 14944-CELSR2-cadherin, EGF LAG seven-pass G-type receptor 2 (flamingo homolog, Drosophila) |
| 15824-LOC51619-ubiquitin-conjugating enzyme HBUCE1 | 14952-FXYD5-FXYD domain containing ion transport regulator 5 |
| 15864-TGM3-transglutaminase 3 (E polypeptide, protein-glutamine-gamma-glutamyltransferase) | 14957-CCT5-chaperonin containing TCP1, subunit 5 (epsilon) |
| 15985-NUCB1-nucleobindin 1 | 15023-INHBA-inhibin, beta A (activin A, activin AB alpha polypeptide) |
| 16065-KNTC2-kinetochore associated 2 | 15027-CCND1-cyclin D1 (PRAD1: parathyroid adenomatosis 1) |
| 16118-FCRH1-Fc receptor-like protein 1 | 15028-CIAO1-WD40 protein Ciao1 |
| 16152-SELS-selenoprotein S | 15180-AKAP8L-A kinase (PRKA) anchor protein 8-like |
| 16163-USP34-ubiquitin specific protease 34 | 15229-NUP155-nucleoporin 155kDa |
| 16196-Oxa1l-oxidase assembly 1-like | 15235-MDH2-malate dehydrogenase 2, NAD (mitochondrial) |
| 16249-NPDC1-neural proliferation, differentiation and control, 1 | 15307-BIRC4-baculoviral IAP repeat-containing 4 |
| 16299-SLC17A5-solute carrier family 17 (anion/sugar transporter), member 5 | 15325-DLG5-discs, large homolog 5 (Drosophila) |
| 16403-APPBP2-amyloid beta precursor protein (cytoplasmic tail) binding protein 2 | 15341-ADORA1-adenosine A1 receptor |
| 16538-SLC16A4-solute carrier family 16 (monocarboxylic acid transporters), member 4 | 15354-GRIN1-glutamate receptor, ionotropic, N-methyl D-aspartate 1 |
| 16549-ARHGEF6-Rac/Cdc42 guanine nucleotide exchange factor (GEF) 6 | 15363-CTSE-cathepsin E |
| 16566-TRIM6-tripartite motif-containing 6 | 15416-ADD1-adducin 1 (alpha) |
| 16592-ASGR2-asialoglycoprotein receptor 2 | 15422-PFN2-profilin 2 |
| 16780-CAPS-calcyphosine | 15435-PLEKHA4-pleckstrin homology domain containing, family A (phosphoinositide binding specific) member 4 |
| 16786-CPB1-carboxypeptidase B1 (tissue) | 15440-SQRDL-sulfide quinone reductase-like (yeast) |
| 16850-PLXND1-plexin D1 | 15454-CHST5-carbohydrate (N-acetylglucosamine 6-O) sulfotransferase 5 |
| 16898-TPD52L1-tumor protein D52-like 1 | 15461-DOK2-docking protein 2, 56kDa |
| 16918-TUBG1-tubulin, gamma 1 | 15488-MGC4083-tubulin beta MGC4083 |
| 16947-HPCA-hippocalcin | 15562-CDC25B-cell division cycle 25B |
| 16997-CPE-carboxypeptidase E | 15677-SYN1-synapsin I |
| 17216-ARG2-arginase, type II | 15699-PES1-pescadillo homolog 1, containing BRCT domain (zebrafish) |

| | |
|---|---|
| 17304-QPRT-quinolinate phosphoribosyltransferase (nicotinate-nucleotide pyrophosphorylase (carboxylating)) | 15753-EEF1E1-eukaryotic translation elongation factor 1 epsilon 1 |
| 17312-8D6A-8D6 antigen | 15754-ZFP91-zinc finger protein 91 homolog (mouse) |
| 17349-MGAT4A-mannosyl (alpha-1,3-)-glycoprotein beta-1,4-N-acetylglucosaminyltransferase, isoenzyme A | 15822-UBA52-ubiquitin A-52 residue ribosomal protein fusion product 1 |
| 17352-JFC1-NADPH oxidase-related, C2 domain-containing protein | 15824-LOC51619-ubiquitin-conjugating enzyme HBUCE1 |
| 17426-DAG1-dystroglycan 1 (dystrophin-associated glycoprotein 1) | 15828-FBXO32-F-box only protein 32 |
| 17464-FLJ30092-AF-1 specific protein phosphatase | 15862-SDHB-succinate dehydrogenase complex, subunit B, iron sulfur (Ip) |
| 17485-RHOA-ras homolog gene family, member A | 16074-HLA-DPB1-major histocompatibility complex, class II, DP beta 1 |
| 17608-ZIC4-Zic family member 4 | 16083-CCNK-cyclin K |
| 17620-EI24-etoposide induced 2.4 mRNA | 16166-RHAG-Rhesus blood group-associated glycoprotein |
| 17668-PSME2-proteasome (prosome, macropain) activator subunit 2 (PA28 beta) | 16350-PSMD3-proteasome (prosome, macropain) 26S subunit, non-ATPase, 3 |
| 17745-LHPP-phospholysine phosphohistidine inorganic pyrophosphate phosphatase | 16399-DNAJB12-DnaJ (Hsp40) homolog, subfamily B, member 12 |
| 17754-PLCG2-phospholipase C, gamma 2 (phosphatidylinositol-specific) | 16469-NFKB1-nuclear factor of kappa light polypeptide gene enhancer in B-cells 1 (p105) |
| 17790-CLEC2-C-type lectin-like receptor-2 | 16860-API5-apoptosis inhibitor 5 |
| 17800-P4HB-procollagen-proline, 2-oxoglutarate 4-dioxygenase (proline 4-hydroxylase), beta polypeptide (protein disulfide isomerase; thyroid hormone binding protein p55) | 16872-NOTCH4-Notch homolog 4 (Drosophila) |
| 17836-MCAM-melanoma cell adhesion molecule | 16947-HPCA-hippocalcin |
| 17862-BBS2-Bardet-Biedl syndrome 2 | 17093-HLA-DRB3-major histocompatibility complex, class II, DR beta 3 |
| 17882-ATP5F1-ATP synthase, H+ transporting, mitochondrial F0 complex, subunit b, isoform 1 | 17283-FGG-fibrinogen, gamma polypeptide |
| 17924-SLC35E1-solute carrier family 35, member E1 | 17426-DAG1-dystroglycan 1 (dystrophin-associated glycoprotein 1) |
| 17934-NTAN1-N-terminal asparagine amidase | 17496-MSR1-macrophage scavenger receptor 1 |
| 17948-hIAN6-human immune associated nucleotide 6 | 17524-TIMP3-tissue inhibitor of metalloproteinase 3 (Sorsby fundus dystrophy, pseudoinflammatory) |
| 17971-LOXL1-lysyl oxidase-like 1 | 17[illegible]R2-transferrin receptor 2 |
| 18072-MEF2B-MADS box transcription enhancer factor 2, polypeptide B (myocyte enhancer factor 2B) | 17662-SUI1-putative translation initiation factor |
| 18162-PTGS1-prostaglandin-endoperoxide synthase 1 (prostaglandin G/H synthase and cyclooxygenase) | 17679-RELB-v-rel reticuloendotheliosis viral oncogene homolog B, nuclear factor of kappa light polypeptide gene enhancer in B-cells 3 (avian) |
| 18170-RPA3-replication protein A3, 14kDa | 17936-FADS1-fatty acid desaturase 1 |
| 18225-CFL1-cofilin 1 (non-muscle) | 17948-hIAN6-human immune associated |

| | |
|---|---|
| | nucleotide 6 |
| 18228-FBN1-fibrillin 1 (Marfan syndrome) | 17963-RAF1-v-raf-1 murine leukemia viral oncogene homolog 1 |
| 18389-SOX7-SRY (sex determining region Y)-box 7 | 18072-MEF2B-MADS box transcription enhancer factor 2, polypeptide B (myocyte enhancer factor 2B) |
| 18443-STK35-serine/threonine kinase 35 | 18210-FCGR2B-Fc fragment of IgG, low affinity IIb, receptor for (CD32) |
| 18464-ZFYVE20-zinc finger, FYVE domain containing 20 | 18212-CENPF-centromere protein F, 350/400ka (mitosin) |
| 18544-PACSIN1-protein kinase C and casein kinase substrate in neurons 1 | 18228-FBN1-fibrillin 1 (Marfan syndrome) |
| 18565-GNB5-guanine nucleotide binding protein (G protein), beta 5 | 18470-KRT13-keratin 13 |
| 18594-USP37-ubiquitin specific protease 37 | 18482-DLC1-deleted in liver cancer 1 |
| 18623-SOX10-SRY (sex determining region Y)-box 10 | 18509-SIAT7D-sialyltransferase 7D ((alpha-N-acetylneuraminyl-2,3-beta-galactosyl-1,3)-N-acetyl galactosaminide alpha-2,6-sialyltransferase) |
| 18648-CL640-hypothetical protein CL640 | 18648-CL640-hypothetical protein CL640 |
| 18686-SET-SET translocation (myeloid leukemia-associated) | 18758-MACF1-microtubule-actin crosslinking factor 1 |
| 18751-MCCC1-methylcrotonoyl-Coenzyme A carboxylase 1 (alpha) | 18761-ch-TOG-KIAA0097 gene product |
| 18798-AMT-aminomethyltransferase (glycine cleavage system protein T) | 18850-SNTA1-syntrophin, alpha 1 (dystrophin-associated protein A1, 59kDa, acidic component) |
| 18913-SKP2-S-phase kinase-associated protein 2 (p45) | 18851-TRAF2-TNF receptor-associated factor 2 |
| 18916-SERPINE1-serine (or cysteine) proteinase inhibitor, clade E (nexin, plasminogen activator inhibitor type 1), member 1 | 19017-JAZF1-juxtaposed with another zinc finger gene 1 |
| 18950-ACTA2-actin, alpha 2, smooth muscle, aorta | 19142-PRKAR2A-protein kinase, cAMP-dependent, regulatory, type II, alpha |
| 18986-CDC42EP1-CDC42 effector protein (Rho GTPase binding) 1 | 19154-TRIAD3-TRIAD3 protein |
| 19008-ECH1-enoyl Coenzyme A hydratase 1, peroxisomal | 19171-C1S-complement component 1, s subcomponent |
| 19191-IGJ-immunoglobulin J polypeptide, linker protein for immunoglobulin alpha and mu polypeptides | 19190-HLA-DRB3-major histocompatibility complex, class II, DR beta 3 |
| 19199-FURIN-furin (paired basic amino acid cleaving enzyme) | 19199-FURIN-furin (paired basic amino acid cleaving enzyme) |
