# Supplementary material for: Prediction of Graft-Versus-Host Disease in Humans by Donor Gene-Expression Profiling
Source: PLoS Med. 2007 Jan 30;4(1):e23. doi: 10.1371/journal.pmed.0040023 (PMC1796639; doi:10.1371/journal.pmed.0040023)
Supplement: Alternative Language Abstract S1 — (26 KB DOC) [file pmed.0040023.sd001.doc]

**RÉSUMÉ**

**Contexte Théorique**

Suite à une transplantation de cellules hématopoïétiques allogéniques (CHA), la maladie du greffon contre l’hôte (MGCH) résulte de la reconnaissance des antigènes d’histocompatibilité du receveur par les lymphocytes T du donneur. Fait important, l’histoincompatibilité donneur/receveur est nécessaire mais non suffisante pour déclencher une MGCH. Dans cette perspective, le but de notre travail était d’évaluer si certains donneurs étaient de plus forts “allorépondeurs” que d’autres, et ainsi plus susceptibles de causer une MGCH.

**Devis Expérimental et Résultats**

Nous avons étudié le profil d’expression génique des lymphocytes T CD4 et CD8 de 50 donneurs de CHA au moyen de biopuces à ADN. Nous avons trouvé que le profil d’expression génique était différent chez les donneurs qui ont induit ou non une MGCH chez leur receveur. De plus, des études statistiques détaillées de données obtenues par réaction de polymérase en chaîne quantitative ont démontré que le phénotype “donneur dangereux” (MGCH+) était un trait polygénique déterminé notamment par des gènes impliqués dans la prolifération cellulaire et la voie de signalisation TGF-β.

**Conclusions et Implications**

Nos résultats suggèrent fortement que le profil d’expression génique des lymphocytes T du donneur a une influence déterminante sur la survenue d’une MGCH suite à une transplantation de CHA. La capacité d’identifier les forts et faibles allorépondeurs pourrait se révéler d’une grande utilité en de transplantation de CHA et éventuellement en greffe d’organe.
